# ATP regeneration-driven biocatalytic production of 1,12-dodecanediol with improved cofactor supply

**DOI:** 10.1186/s40643-026-01069-6

**Published:** 2026-05-25

**Authors:** Yoon Jung Jung, Gaeul Kim, Kyungjae Yu, Byung Wook Lee, Jung Bin Shin, Jung-Oh Ahn, Si Jae Park, See-Hyoung Park, Hyun Gi Koh, Kyungmoon Park

**Affiliations:** 1 Department of Biological and Chemical Engineering, Hongik University, Sejong, 30016 Republic of Korea; 2 Biotechnology Process Engineering Center, Korea Research Institute of Bioscience and Biotechnology, Ochang, 28116 Republic of Korea; 3 Graduate Program in System Health Science and Engineering, Department of Chemical Engineering and Materials Science, Ewha Womans University, Seoul, 03760 Republic of Korea

**Keywords:** 1,12-Dodecanediol, Biocatalytic conversion, Carboxylic acid reductase, ATP regeneration, Polyphosphate kinase (PPK2)

## Abstract

1,12-Dodecanediol is a high-value chemical widely used in the polymer and fine chemical industries. However, its industrial production is currently based on petroleum-derived processes associated with high energy consumption and environmental concerns. To address these limitations, microbial bioconversion of 1,12-dodecanedioic acid (C12 diacid) to 1,12-dodecanediol (C12 diol) has been investigated as a sustainable production route. In this pathway, carboxylic acid reductase (CAR) requires ATP and NADPH as essential cofactors, making efficient cofactor supply an important factor for achieving high conversion efficiency. In this study, a recombinant *Escherichia coli*–based biocatalytic system was developed to enhance C12 diol production through ATP regeneration. ATP availability was identified as an important factor for whole-cell bioconversion, and a polyphosphate kinase 2 (PPK2) from *Erysipelotrichaceae bacterium* was introduced to regenerate ATP from AMP, increasing the conversion yield from 22.0% to 31.8% at 40 mM substrate. Supplementation with sodium hexametaphosphate (SHMP, polyP_6_) further improved diol production, reaching 19.5 mM C12 diol with a conversion yield of 48.8%. In the cell-lysate-based conversion, NADPH and NADH exhibited comparable conversion efficiencies up to 70 mM MgCl_2_. Diol production was influenced by MgCl_2_ concentration, with the highest conversion obtained at 100 mM MgCl_2_ in the presence of NADPH, yielding approximately 28 mM C12 diol (~ 70% conversion) within 1 h. Overall, this study highlights the importance of ATP regeneration and cofactor supply for improving C12 diol production.

**Graphical abstract:**

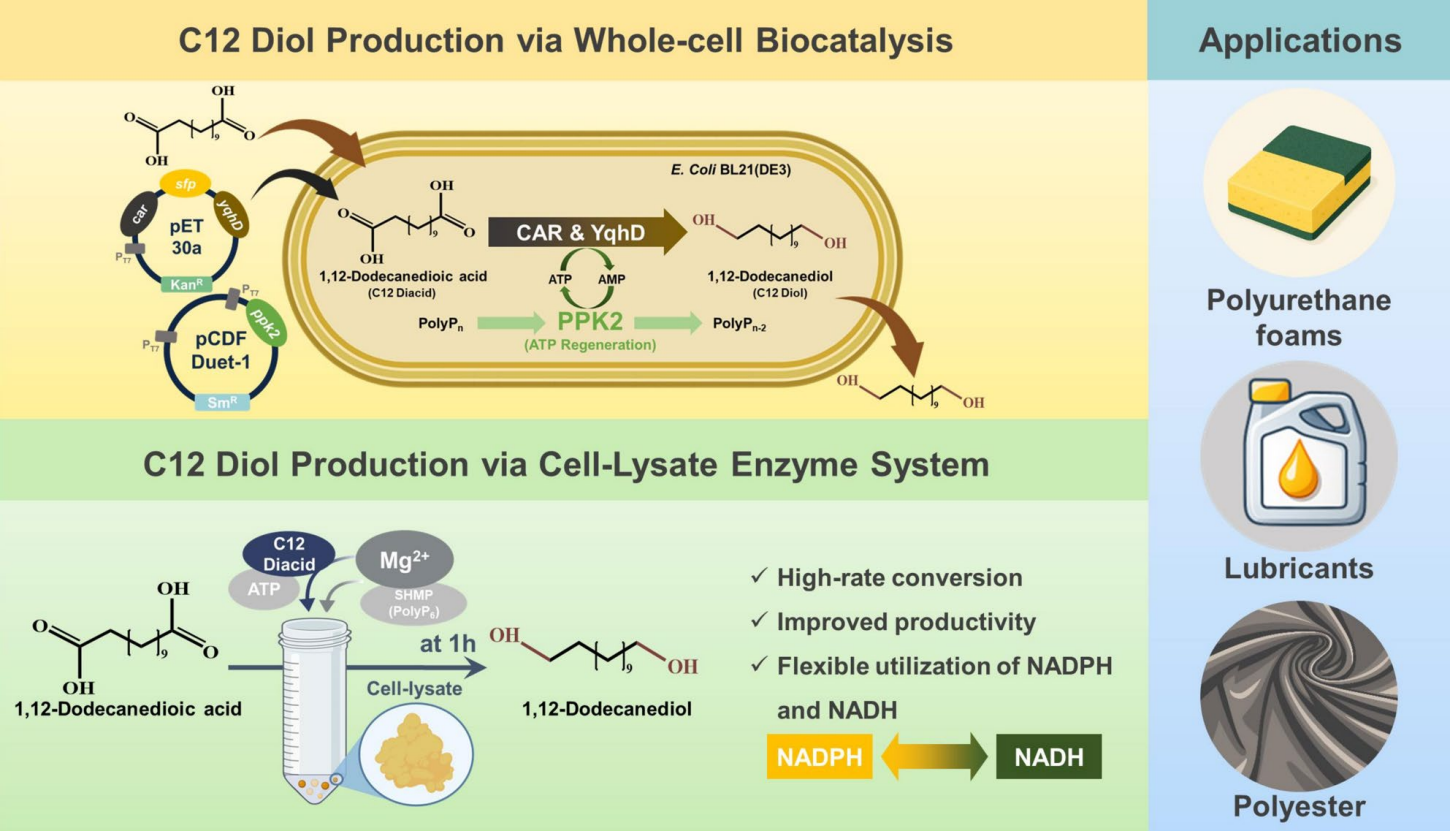

**Supplementary Information:**

The online version contains supplementary material available at 10.1186/s40643-026-01069-6.

## Introduction

1,12-Dodecanediol is a valuable long-chain aliphatic diol consisting of a linear twelve-carbon backbone with terminal hydroxyl groups. Due to its unique molecular structure, it serves as an important building block and versatile chemical intermediate for synthesizing high-performance polymers, particularly polyesters and polyurethanes (Ahsan et al. 2018; Hsieh et al. 2018). The elongated and flexible aliphatic chain of 1,12-dodecanediol provides enhanced thermo-mechanical properties, improved chemical resistance, and lower water absorption to the resulting materials compared to those derived from short-chain diols (Stempfle et al. 2016). Additionally, its notable latent heat and phase transition characteristics near 80 °C ℃ have attracted interest for its use as a phase-change material (PCM) in thermal energy storage systems, such as solar harvesting and waste-heat recovery (Ensari and Alkan 2025). Medium‑ and long‑chain α,ω‑alkanediols, including 1,12-dodecanediol, are used in personal care formulations, industrial lubricants, and specialty polymer precursors for medical and high-value applications (Hsieh et al. 2018).

Currently, industrial production of 1,12-dodecanediol primarily relies on petroleum-derived chemical processes. These conventional methods typically require multi-step oxidation and reduction sequences conducted under demanding conditions, such as elevated temperatures and pressures, often mediated by metal catalysts. Although these processes allow for large-scale production, they are often associated with high energy intensity, reliance on fossil feedstocks, and significant environmental impacts from catalyst-related waste and byproduct formation. In response to these sustainability challenges, there is a growing interest in developing robust biocatalytic frameworks as eco-friendly alternatives to traditional chemical synthesis (Ahsan et al. 2018; Lu et al. 2024; Schaffer and Haas 2014).

Various biocatalytic strategies for 1,12-dodecanediol production have been explored using diverse substrates such as omega-hydroxy fatty acids, fatty acids, and dicarboxylic acids (Lu et al. 2024; Lu and Weusthuis 2025). Among these, multi-enzyme cascades integrating CYP153 monooxygenases with carboxylic acid reductase (CAR) have emerged as widely used bioconversion pathways (Ahsan et al. 2018; Hsieh et al. 2018; Park et al. 2022). Specifically, the CAR derived from *Mycobacterium abscessus* (Mab4714) has demonstrated superior catalytic performance and broad substrate specificity toward long-chain aliphatic dicarboxylic acids, making it an effective candidate for producing terminal diols (Cha et al. 2021; Khusnutdinova et al. 2017). For the subsequent reduction step, YqhD was selected based on our previously published comparative evaluation of aldehyde reductases, in which YqhD showed robust performance relative to alternative candidates (e.g., YahK and YihU) under ATP-demanding whole-cell bioconversion conditions (Lee et al. 2025). Previous laboratory-scale studies using whole-cell platforms have generally reported 1,12-dodecanediol titers within a limited range of low to several tens of millimolar (Ahsan et al. 2018; Kim et al. 2025). While higher titers have been achieved through fed-batch fermentation, such processes typically require cultivation periods exceeding 48 h, which lowers volumetric productivity and complicates process intensification (Hsieh et al. 2018; Park et al. 2025).

A major challenge in these biocatalytic processes is the significant difference in reported productivities across studies, even when using similar CAR-mediated pathways (Hsieh et al. 2018; Khusnutdinova et al. 2017). Recent evidence suggests that these variations are more closely linked to the efficiency of cofactor supply and regeneration rather than to the basic design of the metabolic pathways (Akhtar et al. 2013 a; Kramer et al. 2020). The catalytic activity of CAR is highly dependent on the availability of both ATP and NADPH (Horvat and Winkler 2020; Karava et al. 2025; Strohmeier et al. 2020). Given the high cost and continuous consumption of these cofactors, considerable research efforts have been directed toward the development of efficient ATP and NADPH regeneration systems to enhance both the economic viability and overall productivity of CAR-mediated bioprocesses (Chen and Zhang 2021; Horvat and Winkler 2020; Strohmeier et al. 2020). Furthermore, the CAR-mediated reaction involves an ATP-dependent adenylation step that requires divalent metal ions, specifically Mg^2+^, to stabilize the enzyme-bound ATP complex and facilitate catalysis (Basri et al. 2023 a; Cha et al. 2021). This requirement for Mg^2+^ is also shared by ATP regeneration enzymes such as polyphosphate kinase 2 (PPK2), which uses the metal ion to facilitate the transfer of phosphoryl groups from polyphosphate to nucleotides (Cao et al. 2018; Motomura et al. 2014; Neville et al. 2022). Despite the importance of these cofactors and metal ions, systematic evaluations of different reaction environments, specific regeneration systems, and the optimization of magnesium concentrations remain relatively limited for 1,12-dodecanediol production. Moreover, most existing studies rely on whole-cell platforms that face inherent mass transfer limitations of long-chain substrates (Ahsan et al. 2018; Cha et al. 2021). These limitations often result in prolonged reaction times and limited volumetric productivities, posing a significant bottleneck for industrial feasibility. Thus, optimizing the reaction environment to both ensure efficient cofactor regeneration and overcome cell membrane barriers is crucial for achieving high-rate bioconversion.

In this study, we developed a high-rate biocatalytic platform designed to overcome the kinetic and economic bottlenecks of 1,12-dodecanediol production using engineered *Escherichia coli*. While the individual enzymes for this pathway have been previously reported, their systematic integration into a cascade specifically optimized with a PPK2-based ATP regeneration loop provides a practical process-level strategy for overcoming energetic bottlenecks. An ATP regeneration system was introduced to support the energy demand of the reaction, and whole-cell and cell-lysate formats were compared to establish a rapid format for diol production. Crucially, the system was optimized not only for metal ion conditions but also for the utilization of NADH as a more cost-effective and stable alternative to NADPH, thereby enhancing the economic viability of the process. Under the optimized conditions, a conversion of 69.4% was achieved within a shortened reaction time, resulting in a productivity of 5.60 g/L/h, among the highest values reported for flask-scale 1,12-dodecanediol bioconversion. Overall, this work presents an accelerated route for 1,12-dodecanediol production and provides practical guidance for improving cofactor-dependent long-chain diol bioconversion.

## Materials and methods

### Chemicals

Chemicals used in this study included dodecanedioic acid (99%), ammonium hydroxide solution, β-nicotinamide adenine dinucleotide phosphate (NADPH; ≥93%), β-nicotinamide adenine dinucleotide (NADH; 98%), potassium phosphate dibasic (≥ 98.0%), potassium phosphate monobasic (≥ 98.0%), sodium hexametaphosphate (96%), isopropyl β-D-1-thiogalactopyranoside (IPTG; ≥ 99%), kanamycin, BugBuster^®^ Master Mix, *N*,* O*-bis(trimethylsilyl)trifluoroacetamide (BSTFA; ≥ 99.0%), 12-hydroxydodecanoic acid (97%) and 1,12-dodecanediol, which were purchased from Sigma-Aldrich (St. Louis, MO, USA). Spectinomycin was purchased from BioShop (Burlington, ON, Canada). Tris–HCl buffer (pH 8.5) was obtained from LPS Solutions (Daejeon, Republic of Korea). Adenosine 5′-triphosphate disodium salt hydrate (ATP; >98.0%) was purchased from Tokyo Chemical Industry (Tokyo, Japan). Dextrose anhydrous (99.5%) and magnesium chloride hexahydrate (98%) were obtained from Samchun Chemicals (Seoul, Republic of Korea). Pyridine (99.5%) was purchased from Duksan Chemicals (Republic of Korea).

## Plasmids and bacterial strains

*Escherichia coli* DH5α was used for genetic manipulation, including plasmid construction and cloning procedures, whereas *E. coli* BL21(DE3) was employed for protein expression and preparation of whole-cell and cell-lysate biocatalysts. *E. coli* BL21(DE3) carrying pET-30a(+)::*car*::*sfp*::*yqhD* was used as the biocatalyst in this study. For ATP regeneration, the *ppk2* gene was cloned into the pCDFDuet-1 vector. The pCDFDuet-1 vector was specifically selected to ensure plasmid compatibility with the pET-30a(+) vector; its CloDF13 replicon is fully compatible with the pBR322 origin of pET-30a(+), unlike other vectors such as pETDuet-1 that share the same origin. The pCDFDuet-1::*ppk2* construct was generated by restriction enzyme–based cloning using primers designed for this study. The *ppk2* gene was inserted into the multiple cloning site 2 (MCS-2) for expression, while the multiple cloning site 1 (MCS-1) was left vacant for potential future insertion of additional genes. The nucleotide sequence of the *ppk2* gene and all primer sequences used for cloning in this study are provided in Supplementary Table S1 (Additional file 1). The resulting plasmids were transformed into *E. coli* BL21(DE3) for subsequent whole-cell and cell-lysate-based bioconversion experiments.

### Cell cultivation and preparation for bioconversion

For enzyme expression and biocatalyst preparation, *Escherichia coli* strains listed in Table 1 were used. Pre-cultures were prepared by inoculating single colonies into 4 mL of LB medium and incubating at 37 °C with shaking at 200 rpm for 12–13 h. The pre-cultures were then inoculated into 100 mL of fresh LB medium at an inoculation ratio of 4% (v/v). Cells were cultivated for approximately 24 h at 200 rpm, with incubation at 37 °C until IPTG induction (OD_600_ = 0.6–0.8), followed by continued cultivation at 30 °C after induction. Protein expression was induced by the addition of isopropyl β-D-1-thiogalactopyranoside (IPTG) to a final concentration of 0.05 mM. Cells were harvested by centrifugation at 4 °C and 4,000 rpm for 20 min using 50 mL Falcon conical tubes and subsequently processed for whole-cell or cell-lysate-based bioconversion as described below. Reaction mixtures for bioconversion contained 100 mM potassium phosphate buffer (pH 7.5), dodecanedioic acid (10 or 40 mM), MgCl_2_ (0–130 mM), ATP (0–20 mM), NAD(P)H (0–8 mM), sodium hexametaphosphate (SHMP; 0–30 mM), and glucose (1%, w/v). The pH of the reaction mixture was adjusted to 7.5 using 5 N HCl or 5 N NaOH, and the final reaction volume was adjusted with distilled deionized water (DDW).

**Table 1:** List of strains and plasmids used in this study

Strains and plasmids	Genotype of strain and plasmid	Reference or source
Strains		
*E.coli* DH5α	F- *Φ80lacZΔM15 Δ(lacZYA-argF)U169 deoR recA1 endA1 hsdR17(r*_*k*_^*−*^,*m*_*k*_^*+*^*)phoAsupE44thi-1gyrA96relA1* ​	Enzynomics
*E.coli* BL21(DE3)	F-dcm ompT hsdS(rB- mB-) gal (DE3)	Enzynomics
*E.coli* C	*E.coli* BL21(DE3) harboring pET-30a(+)::*car*::*sfp*::*yqhD*	This study
*E.coli* CP	*E.coli* BL21(DE3) harboring pET-30a(+)::*car*::*sfp*::*yqhD* and pCDFDuet-1::*ppk2*(MCS2)	This study
Plasmids		
pET30a(+)::*car*::*sfp*::*yqhD*	pET30a derivative; *car*, *sfp*, *yqhD*, Km^R^	This study
pCDFDuet-1::*ppk2*(MCS2)	pCDFDuet-1 derivative; *ppk2*, Sm^R^	This study

### Analysis of protein expression and solubility

To analyze protein expression and solubility, cells adjusted to an OD_600_ of 20 were harvested, washed, and resuspended in 1 M Tris buffer. Cell disruption was performed using an Ultrasonic Processor (VC 505, Sonics, USA) at 40% amplitude for 2 min on ice. Following lysis, a 20 µL aliquot was collected as the total protein fraction. The remaining lysate was centrifuged (13,000 rpm, 1 min, 4 °C) to separate the soluble and insoluble protein fractions. The pellet was resuspended in an equal volume of Tris buffer for the insoluble fraction. All samples were mixed with 5X Blue SDS Loading Buffer and denatured at 95 °C for 5 min. SDS-PAGE was conducted using 10% Mini-PROTEAN TGX Gels (Bio-Rad, USA), NEWRun SDS Run Buffer (Jubiotech, Korea), and a Color Prestained Protein Standard (10–250 kDa, New England Biolabs, USA). Samples were run at 20–30 mA using a Mini-PROTEAN Tetra Cell (Bio-Rad, USA). Gels were stained with Coomassie Brilliant Blue R-250 (Bio-Rad, USA) for 1 h and destained for 2 h. Finally, the gels were immersed in distilled water overnight for visualization and analysis.

### Whole-cell bioconversion

For whole-cell bioconversion, harvested cell pellets were washed twice with 1 M Tris buffer (pH 8.0) and resuspended in the prepared reaction mixture (Lee et al. 2025). The reactions were carried out at 30 °C with shaking at 200 rpm for 24 h, corresponding to a cell density of OD_600_ = 20. Samples were collected at 6 h intervals for subsequent analysis.

### Cell-lysate-based bioconversion

For cell-lysate preparation, harvested cells were washed once with 1 M Tris buffer (pH 8.0) and centrifuged again to remove the supernatant. The wet cell weight was measured, and cell disruption was performed using BugBuster^®^ Master Mix according to the manufacturer’s protocol. Briefly, 500 µL of BugBuster^®^ Master Mix was added per 0.1 g of wet cell weight, followed by resuspension and incubation at 20 °C for 15–20 min to achieve cell lysis. For cell-lysate-based bioconversion, the prepared reaction mixture was adjusted to pH 7.5 prior to the addition of the cell lysate. After addition of the lysate, the pH was readjusted if necessary, and the final reaction volume was adjusted with DDW. The reactions were conducted in 50 mL Falcon conical tubes with a working volume of 5 mL at 30 °C and 200 rpm, corresponding to an equivalent cell density of OD_600_ = 20. Samples were collected at 0, 1, 2, 4, and 6 h for analysis.

### Sample preparation and GC analysis

To ensure analytical accuracy, tetradecane was added as an internal standard.Samples were dried using a SpeedVac (CVE-2000, EYELA, Tokyo Rikakikai Co., Tokyo, Japan) concentrator and derivatized as described previously with minor modifications (Cha et al. 2021). Briefly, dried samples were treated with a 1:1 (v/v) mixture of N, O-bis(trimethylsilyl)trifluoroacetamide (BSTFA) and pyridine and incubated at 70 °C with shaking at 1,050 rpm for 1 h. Derivatized samples were filtered and analyzed by GC FID (Shimadzu, Japan) using an HP-5ms capillary column (30 m × 0.25 mm × 0.25 μm, Agilent Technologies) with helium as the carrier gas. The injector and detector temperatures were set to 260 °C and 350 °C, respectively. The oven temperature was initially held at 40 °C for 2 min, increased to 70 °C at a rate of 20 °C/min with a 1 min hold, and subsequently ramped to 320 °C at 10 °C/min with a final hold of 5 min.

### Statistical analysis

All experiments were performed in at least three independent experiments (*n* = 3), and the results are presented as the mean ± standard deviation (SD). Statistical analyses were conducted using SigmaPlot 15.0 (Systat Software, Inc., San Jose, CA, USA). For Fig. 3a, a two-tailed Student’s t-test was used to compare the means of two groups. For Figs. 3b, and 5a, b a two-way analysis of variance (ANOVA) was performed to evaluate the effects of independent variables and their interactions, followed by the Holm-Sidak post-hoc test for multiple pairwise comparisons. A *P*-value of less than 0.05 was considered statistically significant (*P < 0.05, **P < 0.01, and ***P < 0.001). To maintain visual clarity, non-significant differences (*P* ≥ 0.05) are not explicitly indicated in the figures.

## Results and discussion

### Establishment of the biocatalytic pathway and evaluation of ATP availability

**Fig. 1:**
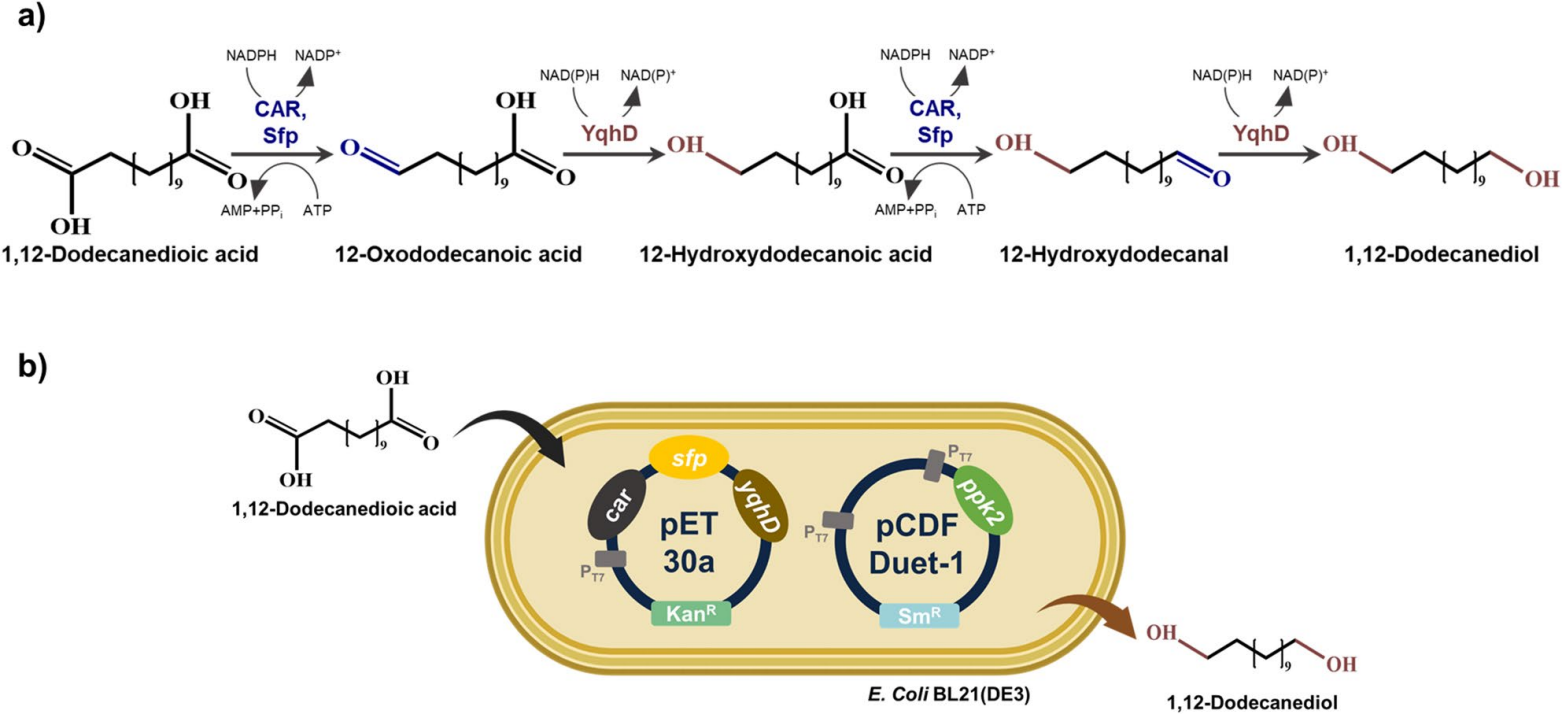
Biocatalytic conversion of 1,12-dodecanedioic acid to 1,12-dodecanediol. **a **The biocatalytic pathway for the step-wise reduction of 1,12-dodecanedioic acid to 1,12-dodecanediol. The *E. coli* NADPH-dependent alcohol dehydrogenase YqhD was employed for the aldehyde reduction step. Abbreviations: CAR, carboxylic acid reductase; Sfp, phosphopantetheinyl transferase. **b** Schematic representation of the recombinant *E. coli* BL21(DE3) whole-cell system. The host strain harbors two plasmids: pET30a-expressing *car*, *sfp*, and *yqhD*, and pCDF Duet-1-expressing *ppk2* for cofactor regeneration

To develop an efficient bioconversion route for 1,12-dodecanediol, a biocatalytic cascade was designed to convert 1,12-dodecanedioic acid to 1,12-dodecanediol through sequential reduction reactions mediated by carboxylic acid reductase (CAR) from *Mycobacterium abscessus* (Mab4714) and NADPH-dependent alcohol dehydrogenase (YqhD) from *Escherichia coli* (Fig. 1a). The soluble expression of CAR, Sfp, YqhD, and PPK2 in the recombinant *E. coli* strains was confirmed by SDS-PAGE analysis (Supplementary Figure S2). Although preliminary screening of three different CAR candidates (Mab2962, Mab4714, and MsCAR) showed comparable catalytic performance toward the Dodecanedioic acid (Supplementary Figure S3, Supplementary Table S2), Mab4714 was prioritized due to its well-documented reliability for long-chain substrates and its robust expression stability in our platform (Cha et al. 2021; Khusnutdinova et al. 2017). Additionally, YqhD was employed as the aldehyde reduction enzyme based on our previously published reductase comparison (Lee et al. 2025). The recombinant *E. coli* system used to implement this pathway is shown in Fig. 1b. In this four-step pathway, CAR catalyzes the ATP-dependent reduction of carboxylic acids to aldehydes, with NAD(P)H serving as the essential reducing equivalent for the overall process. Notably, CAR is a complex multi-domain enzyme that requires a specific post-translational modification to become catalytically active. This activation is mediated by a phosphopantetheinyl transferase (Sfp), which catalyzes the covalent attachment of a 4’-phosphopantetheine prosthetic group from coenzyme A to a conserved serine residue within the peptidyl carrier protein (PCP) domain of the CAR (Basri et al. 2023 b; Butler and Kunjapur 2020). This modification converts the inactive apo-CAR into its functional holo-form, enabling the tethering and subsequent reduction of the acyl-adenylate intermediate. To ensure this essential transition, Sfp was co-expressed in our engineered platform, establishing a functional biocatalytic system for efficient C12 diol production.

**Fig. 2:**
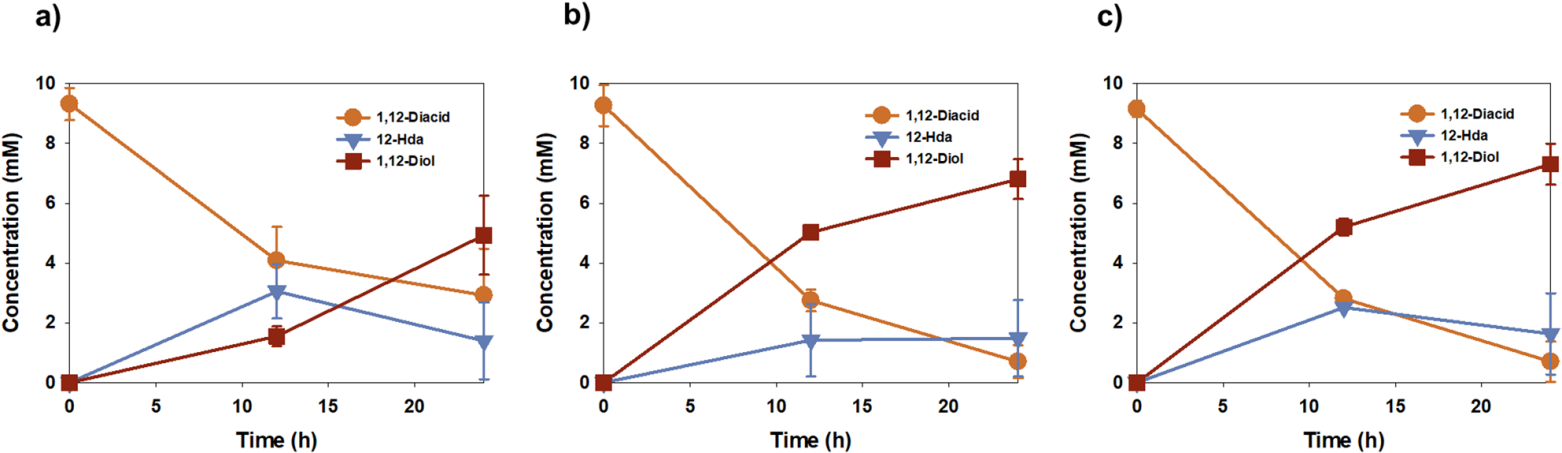
Effect of ATP concentration on whole-cell bioconversion of 1,12-dodecanediol. Time-course profiles of whole-cell bioconversion were monitored using 10 mM of initial substrate in the **a** absence of ATP and in the **b** presence of 10 mM or **c** 20 mM ATP

Given the ATP requirement of the CAR-catalyzed step, whole-cell bioconversion experiments were conducted using 10 mM of initial substrate under varying ATP concentrations to evaluate the effect of external ATP supply on C12 diol production. (Fig. 2). In the absence of exogenous ATP, substrate consumption and diol formation were relatively low, with the 1,12-dodecanediol concentration reaching only 4.8 mM after 24 h (Fig. 2a). In contrast, supplementation with 10 mM ATP increased diol production, reaching about 5 mM after 12 h and approximately 6.8 mM after 24 h (Fig. 2b). Further increasing the ATP concentration to 20 mM resulted in a final titer of 7.6 mM, representing a 1.5-fold increase compared to the condition without ATP supplementation (Fig. 2c).

These results demonstrate that ATP availability is a key factor influencing the efficiency of the CAR-mediated conversion step in whole-cell systems. The observed increase in diol titers with higher ATP concentrations confirms that sufficient energy supply is necessary to achieve improved productivity. This is consistent with the catalytic mechanism of CAR, which requires the stoichiometric consumption of ATP to activate the carboxylic acid substrate via an acyl-adenylate intermediate (Akhtar et al. 2013 b). However, since continuous external supplementation of expensive cofactors is not economically feasible for large-scale applications, these findings highlighted the need for an integrated ATP regeneration system to sustain the bioconversion process efficiently (Andexer and Richter 2015).

### Enhancement of 1,12-dodecanediol production via ATP regeneration using polyphosphate kinase 2

**Fig. 3:**
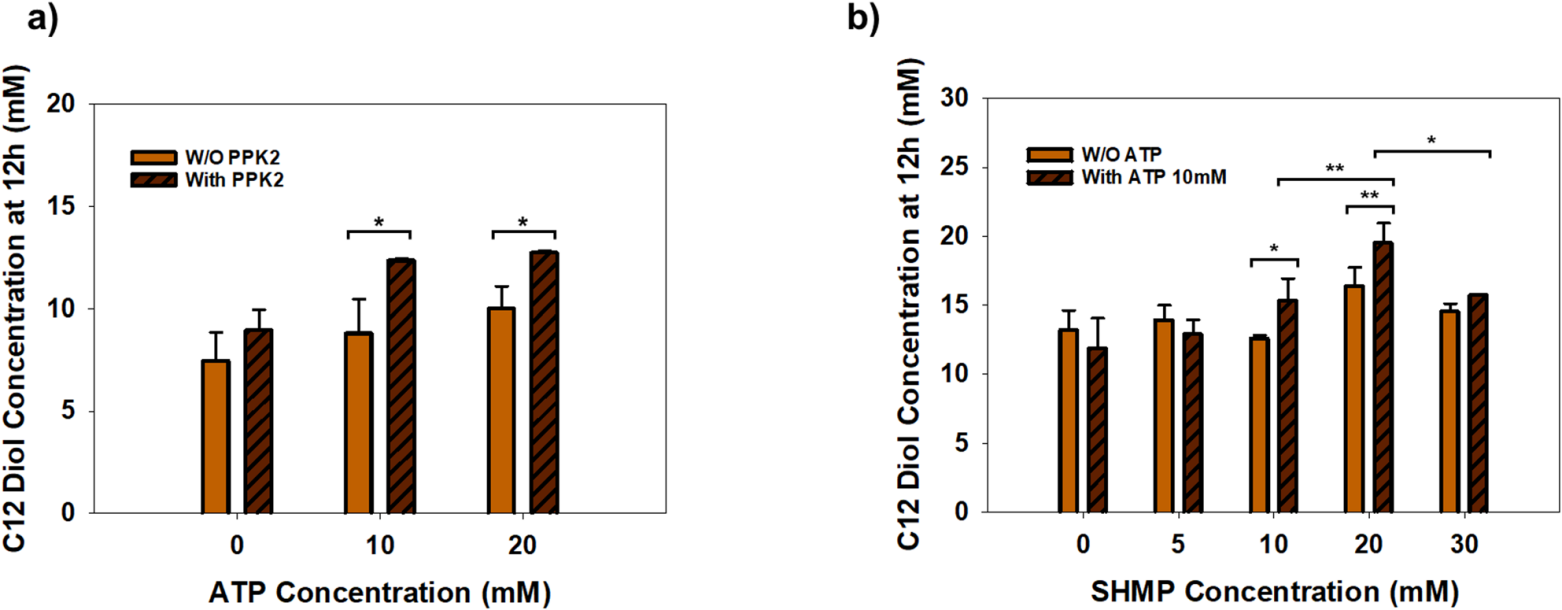
Effect of ATP regeneration on whole-cell bioconversion of 1,12-dodecanediol. **a** The effect of ATP regeneration on 1,12-dodecanediol production was evaluated using 40 mM of initial substrate by comparing whole-cell bioconversion reactions conducted in the absence or presence of polyphosphate kinase (PPK2) at different ATP concentrations (0–20 mM). **b** The influence of sodium hexametaphosphate (SHMP, PolyP₆) concentration on ATP regeneration–driven diol production was examined in the absence or presence of ATP (10 mM). Data are presented as the mean ± SD from at least three independent experiments (*n* = 3). Statistical significance was determined by Student’s t-test (a) or two-way ANOVA followed by Holm-Sidak post-hoc test (b). Asterisks indicate significant differences (*P < 0.05)

To overcome the limitations of external ATP supplementation, a whole-cell bioconversion system was established using an *Escherichia coli* strain expressing polyphosphate kinase 2 (PPK2) from *Erysipelotrichaceae bacterium*. This specific PPK2 was chosen based on prior reports and our comparative evaluation of PPK2-based ATP regeneration under high substrate loading and optimized metal-ion conditions (Tavanti et al. 2021; Yu et al. 2026). The introduction of PPK2 aimed to facilitate the internal regeneration of ATP from AMP, which is generated as a byproduct during the CAR-catalyzed adenylation step. The impact of the ATP regeneration system was first evaluated by comparing diol production in the presence and absence of PPK2 at various ATP concentrations with an increased initial substrate concentration of 40 mM (Fig. 3a). In the absence of PPK2, increasing the external ATP concentration led to a modest improvement in productivity. In particular, the 1,12-dodecanediol titers reached 8.2, 10.1, and 11.2 mM after 12 h for 0, 10, and 20 mM ATP supplementation, respectively (Fig. 3a). This relatively limited increase in productivity likely stems from the low permeability of the *E. coli* cell membrane to phosphorylated cofactors, a well-documented constraint in whole-cell biocatalysis (Heuser et al. 2007; Richter et al. 2010). In contrast, the strain expressing PPK2 exhibited consistently higher diol titers under identical conditions, yielding approximately 12.3 mM with 10 mM ATP and 12.8 mM with 20 mM ATP at the same time point (Fig. 3a). This represented a 20–30% increase in productivity, suggesting that PPK2 effectively regenerates ATP from the AMP byproduct. In this study, the PPK2 system was specifically introduced to address the accumulation of AMP during the CAR reaction. By directly recycling the AMP byproduct, this targeted strategy provides a more controllable and efficient energy supply compared to relying on the cell’s complex central metabolism. From a mechanistic standpoint, the Class III PPK2 from *Erysipelotrichaceae* was selected because it can phosphorylate AMP to ATP using polyphosphate, whereas Class I/II PPK2 enzymes are typically reported to primarily utilize ADP (Tavanti et al. 2021). This AMP-recycling activity is relevant to our system because the CAR-mediated step continuously generates AMP, and recycling AMP helps sustain the adenylate pool and ATP availability during bioconversion. The efficiency of this regeneration system is highly dependent on the availability of polyphosphate as a phosphoryl donor (Motomura et al. 2014). Therefore, diol production was examined at varying concentrations of sodium hexametaphosphate (SHMP; polyP_6_), the substrate for PPK2 (Yu et al. 2026) (Fig. 3b). In the absence of exogenous ATP, increasing the SHMP concentration significantly improved bioconversion efficiency. The highest diol titer of 20.2 mM was achieved at 20 mM SHMP, which was approximately 1.4-fold higher than the titer obtained without SHMP supplementation (Fig. 3b). This improvement confirms that polyphosphate serves as a cost-effective and efficient energy source for driving ATP-demanding biocatalytic processes.

Notably, further increasing the SHMP concentration to 30 mM did not lead to additional improvements in diol production, and the titers remained stagnant or slightly decreased (Fig. 3b). This plateau effect may be attributed to the sequestration of divalent metal ions, particularly Mg^2+^, by high concentrations of polyphosphate. Both CAR and PPK2 generally require divalent metal ions for their catalytic activity, with Mg^2+^ typically serving as the most effective cofactor (Basri et al. 2023 a; Cha et al. 2021; Neville et al. 2022). As reported previously, long-chain polyphosphates can act as potent chelating agents that bind to essential metal ions, which can lead to reduced enzymatic activity (Maier et al. 1999). Consequently, an excessive supply of SHMP can reduce the availability of free Mg^2+^ ions in the reaction medium, thereby negating the benefits of increased phosphoryl donor levels (Kameda et al. 2001; Sun et al. 2021). While some polyphosphate-dependent systems have been reported to tolerate concentrations up to 100 mM (Ding et al. 2024), the optimal concentration is highly dependent on the specific enzyme’s sensitivity to Mg^2+^ availability and the overall reaction conditions. In this study, 30 mM SHMP may represent a range where Mg^2+^ chelation begins to outweigh the benefit of increased phosphoryl donor concentration under our reaction conditions. Overall, these findings demonstrate that the PPK2-based system provides a more efficient and sustainable strategy for ATP supply than direct supplementation, highlighting the importance of precisely optimizing the balance between polyphosphate and essential metal ions to maximize bioconversion efficiency.

### Comparison of whole-cell and cell-lysate biocatalysis for 1,12-dodecanediol production

**Fig. 4:**
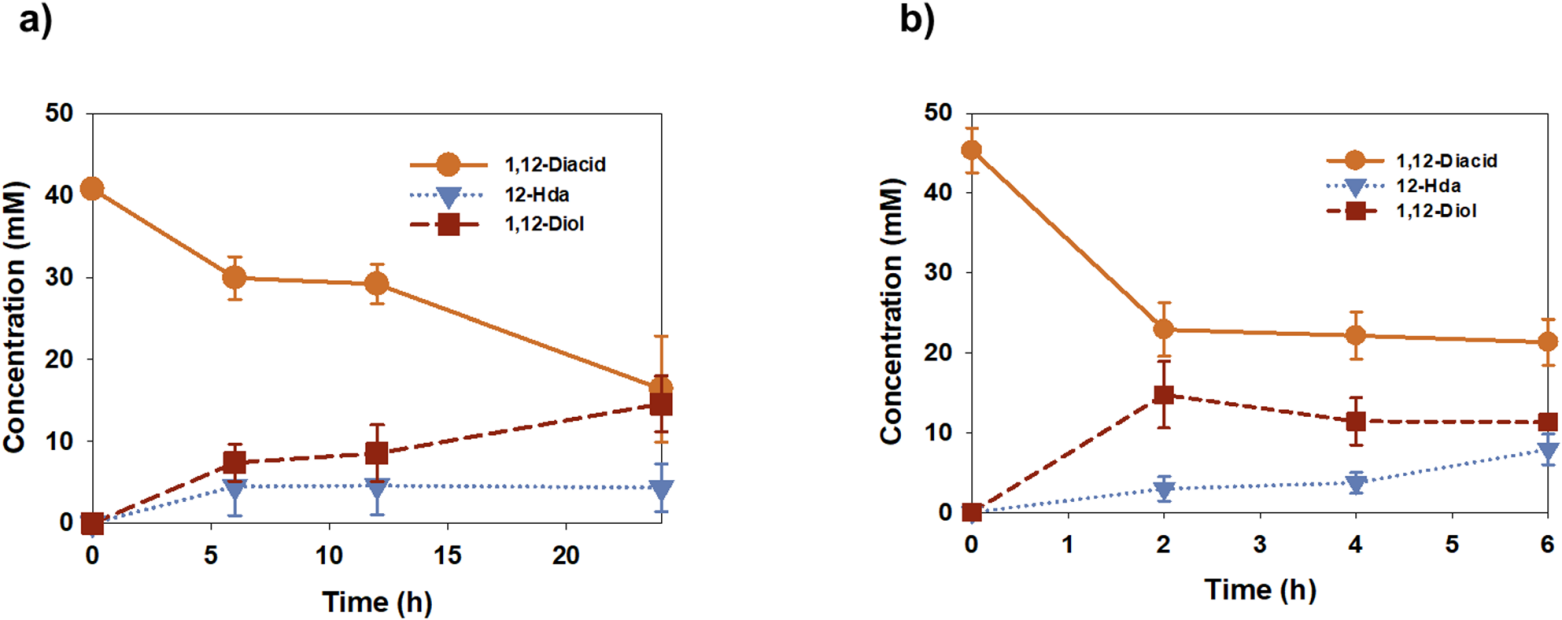
Comparison of whole-cell and cell-lysate biocatalysis for 1,12-dodecanediol production. Time-course profiles of 1,12-dodecanediol production were compared between **a** whole-cell bioconversion and **b** cell-lysate–based enzyme conversion. The concentrations of 1,12-dodecanedioic acid, 12-hydroxydodecanoic acid, and 1,12-dodecanediol were monitored over time

Following the evaluation of the ATP regeneration module, the performance of 1,12-dodecanediol production was compared between whole-cell and cell-lysate-based systems to determine the most efficient reaction environment (Fig. 4). Both systems utilized the same enzyme cascade (CAR and YqhD) and ATP regeneration module (PPK2), with substrate consumption and product formation monitored over a 24 h period.

Under whole-cell bioconversion conditions (Fig. 4a), the substrate was gradually consumed, and 1,12-dodecanediol accumulated to 14.8 mM after 24 h of reaction. Throughout the process, the intermediate 12-hydroxydodecanoic acid (12-HDA) remained at a consistently low concentration (approximately 4.1–4.4 mM). A molar deficiency of approximately 15% was observed in the total mass balance. This discrepancy may be attributed to the transient nature of the 12-hydroxydodecanal intermediate; as aldehydes are readily consumed by numerous endogenous alcohol dehydrogenases in *E. coli* (Richardson et al. 2020), this intermediate likely maintains trace levels that elude standard quantification. Additionally, the inherent hydrophobicity of C12 chains may lead to partial adsorption to the cell membrane or reaction vessel, further contributing to the missing substrate fraction. This slow but steady conversion profile suggests that the overall reaction rate in whole-cell systems is likely constrained due to mass transfer limitations. Mechanistically, the transport of long-chain dicarboxylic acids across the *E. coli* cell membrane often represents a major bottleneck in biocatalysis (Hsieh et al. 2018). The hydrophobicity of the C12 substrate and the limited cell membrane permeability can restrict substrate accessibility to the intracellular enzymes, thereby limiting the effective catalytic rate.

In contrast, the cell-lysate-based system demonstrated a significantly enhanced bioconversion rate during the initial stages of the reaction (Fig. 4b). The system achieved a maximum diol titer of 14.9 mM within just 2 h. This performance is comparable to the titer obtained only after 24 h in the whole-cell system, representing a 12-fold increase in initial volumetric productivity. The elimination of the cell membrane barrier in the lysate system allows for direct interaction between substrates, cofactors, and enzymes, effectively overcoming the mass transfer resistance typical of whole-cell systems.

Furthermore, the cell-lysate system allows for the precise adjustment of ATP and NADPH levels, bypassing the metabolic and transport constraints of whole cells (Hodgman and Jewett 2012). This control is essential for satisfying the high energy demands of the CAR-mediated pathway through direct optimization of the reaction medium (Ling et al. 2025). These results indicate that while whole-cell biocatalysis offers simplicity in terms of process preparation, the cell-lysate-based approach is a more advantageous and efficient platform for the high-rate production of 1,12-dodecanediol. Consequently, the cell-lysate system was adopted as the main platform for subsequent optimization of reducing equivalents and metal ion concentrations.

### Optimization of Mg^2+^ and redox cofactor supply in ATP regeneration–driven biocatalysis

**Fig. 5:**
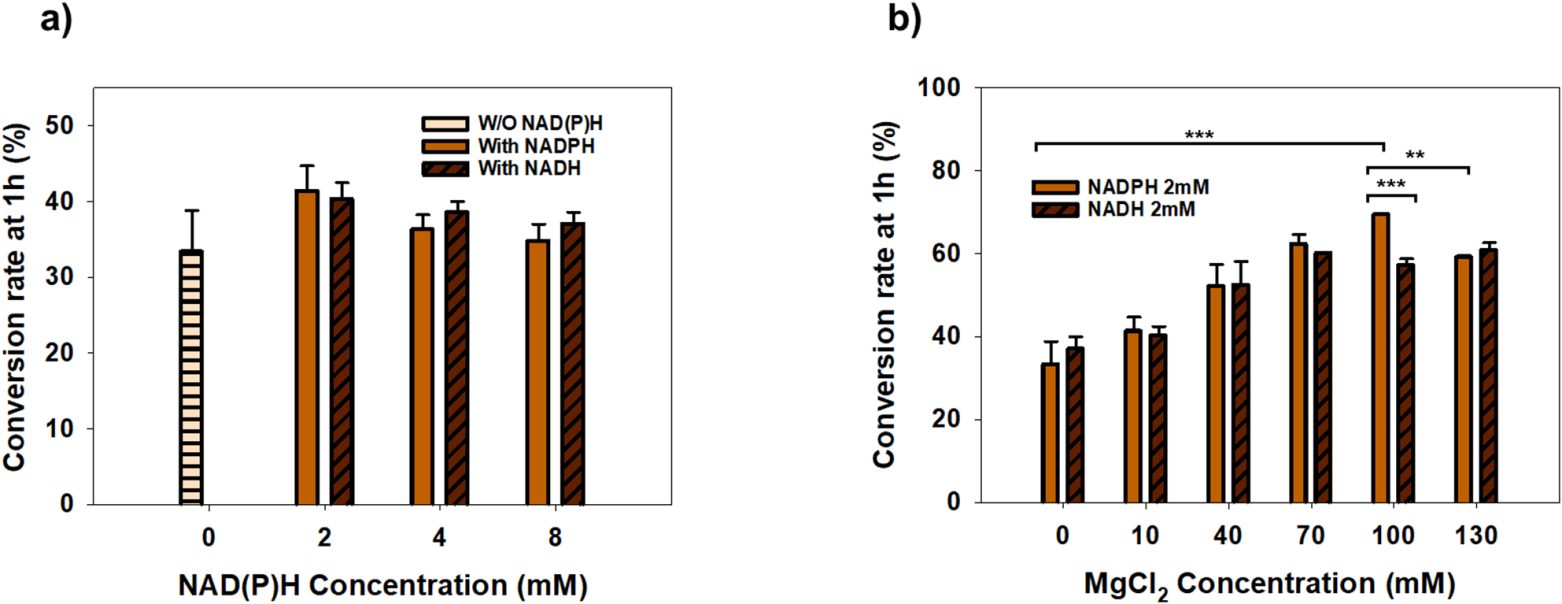
Effect of redox cofactor type and MgCl_2_ concentration on ATP regeneration–driven 1,12-dodecanediol production in enzyme-based conversion. **a** Effect of reducing cofactor type and concentration on 1,12-dodecanediol production was evaluated under ATP regeneration conditions. Bioconversion reactions were performed with either NADPH or NADH supplied at indicated concentrations, while MgCl_2_ concentration was fixed at 10 mM. A control reaction without added NAD(P)H was included for comparison. C12 diol conversion was determined after 1 h of reaction. **b** Comparison of NADPH and NADH as redox cofactors for 1,12-dodecanediol production at various MgCl_2_ concentrations, with the cofactor concentration fixed at 2 mM

Since both CAR and PPK2 require divalent metal ions to maintain their catalytic architecture and stability, the concentration of Mg^2+^ was systematically optimized under the established ATP regeneration conditions (Fig. 5). The 1-hour conversion rate was selected for optimization to evaluate the impact of different parameters during the initial high-activity phase of the multienzyme cascade. Product formation typically approached a plateau within ~ 2 h, which is consistent with time-dependent limitations in the crude lysate environment, including gradual activity loss and/or changes in effective cofactor or metal-ion availability. In this optimization, the Mg^2+^ concentration was prioritized to establish the baseline requirement for enzymatic activity under fixed phosphoryl donor levels. Furthermore, to enhance the economic viability of the process, the feasibility of using NADH was evaluated as a more affordable and stable alternative to NADPH. Although CAR is conventionally classified as an NADPH-dependent enzyme, recent studies have demonstrated its capacity to utilize NADH as an alternative cofactor with comparable conversion levels (Horvat and Winkler 2020; Ling et al. 2025). These findings led to an evaluation of different redox cofactor types and concentrations to maximize conversion efficiency.

The effect of redox cofactor supply was first evaluated by comparing the conversion rates without external supplementation and with varying concentrations of NADPH or NADH (Fig. 5a). In the absence of an external redox cofactor, a baseline conversion rate of approximately 33% was observed, likely supported by endogenous cofactors present in the cell lysate. Supplementation with 2 mM NADPH improved the conversion rate to approximately 41%, while 2 mM NADH yielded a comparable level of around 40% (Fig. 5a). This indicates that YqhD (the NADPH-dependent alcohol dehydrogenase) can utilize both cofactors to facilitate the final reduction step in the lysate system. Since the entire conversion pathway from dodecanoic acid to 1,12-dodecanediol requires a continuous supply of reducing equivalents for both CAR and YqhD, it is important to further consider how the added NADH supports the upstream enzymatic steps, particularly those catalyzed by CAR. Recent structural studies suggest that many CAR enzymes, especially those in the Type I family such as MAB4714, possess a conserved Rossmann-fold domain that provides the structural plasticity to utilize both NADPH and NADH (Horvat and Winkler 2020; Ling et al. 2025). This implies that MAB4714 may directly utilize NADH to facilitate the reduction of the acid substrate, even though NADPH is the conventionally preferred cofactor.

Data represent the mean ± SD (*n* = 3). Statistical significance was determined by two-way ANOVA followed by Holm-Sidak post-hoc test. Significant differences are indicated by asterisks (*P < 0.05, **P < 0.01, ***P < 0.001). Non-significant differences (*P* ≥ 0.05) are not explicitly shown for visual clarity.

Furthermore, the comparable performance observed with NADH in the crude lysate system may also reflect the action of endogenous pyridine nucleotide transhydrogenases, such as UdhA and PntAB, which facilitate cofactor redistribution between NADH and NADPH within the E. coli metabolic network (Clarke et al., 1986 and Sauer et al. 2004). This metabolic interplay likely enables the system to effectively utilize NADH as an indirect reducing equivalent source by channeling reducing power into the NADPH-dependent catalytic steps. Therefore, the high efficiency observed with NADH likely arises from a synergistic effect between possible enzyme-level cofactor flexibility and the metabolic capacity of the host lysate, providing a robust and cost-effective route for long-chain diol production. Nevertheless, increasing the cofactor concentration to 4 mM or 8 mM did not lead to further improvements. Instead, a slight decline was observed, suggesting that excessive concentrations of reducing equivalents may cause inhibitory effects or non-specific interactions that limit the overall flux.

The role of Mg^2+^ was then examined under both NADPH and NADH conditions to address the metal ion demands of the system (Fig. 5b). In the absence of additional MgCl_2_, the conversion rate remained between 33% and 37%. As the MgCl_2_ concentration increased, the conversion rate improved significantly for both cofactors, with a marked increase observed in the 40–70 mM range. The highest conversion level for NADPH (69.4%) was achieved at 100 mM MgCl_2_, while the conversion for NADH reached approximately 60% at 70 mM and remained stable up to 130 mM (Fig. 5b). One possible explanation for the relatively high Mg^2+^ requirement is the sequestration of divalent cations by polyphosphate, which is known to bind metal ions and may decrease the effective concentration of free Mg^2+^ available for PPK2-mediated catalysis (Klompmaker et al. 2017; Nocek et al. 2018). By providing Mg^2+^ in excess of the binding capacity of the polyphosphate (SHMP), sufficient free ions are made available to stabilize the enzyme-ATP complexes of both CAR and PPK2 (Neville et al. 2022). Notably, at 130 mM MgCl_2_, the conversion rate for NADPH showed a slight decline to 59%, possibly due to increased ionic strength or the inhibition of CAR by excessive divalent cations (Müller et al. 2019; Strohmeier et al. 2020).

The efficient production of diol despite low exogenous NAD(P)H supplementation (2 mM) further highlights the significant role of endogenous metabolism in this platform. Control experiments showed that the addition of 1% (w/v) glucose is essential for providing the necessary reducing equivalents (Supplementary Figure S1). While endogenous glucose metabolism provides a baseline NAD(P)H supply, the intentionally designed ATP regeneration system remains a key regulator for optimizing the overall reaction rate (Tang et al. 2021). This metabolic synergy is further enhanced by Mg^2+^ supplementation, which likely accelerates both the target enzymatic activity and the endogenous glycolytic flux by serving as an indispensable cofactor for key enzymes such as hexokinase (Garfinkel and Garfinkel 1985).

Comparing the two cofactors, the Mg^2+^-dependent conversion profiles showed that although NADPH generally provides higher peak conversion, NADH remains a highly effective alternative across a wide range of Mg^2+^ concentrations. From an industrial perspective, the ability to use NADH, which is recognized for its superior stability and significantly lower cost compared to NADPH(Cheng et al. 2025), represents a major advantage for large-scale production.

To compare the performance of the optimized platform, the 1,12-dodecanediol production parameters achieved in this study were compared with previously reported biocatalytic routes (Table 2). Various *Escherichia coli*-based platforms have been developed using substrates such as dodecanoic acid or ω-hydroxy fatty acids. However, most reported systems rely on whole-cell bioconversion and require extended reaction times of 24–48 h to achieve comparable titers (Ahsan et al. 2018; Hsieh et al. 2018). In contrast, the cell-lysate system developed here achieved high-rate bioconversion and reached significant diol concentrations within just 1–2 h. This substantial improvement in volumetric productivity is primarily due to the elimination of mass transfer limitations and the precise optimization of cofactor and metal ion availability. These findings demonstrate that balancing ATP regeneration with optimized Mg^2+^ levels and utilizing NADH-based strategies provide a practical and cost-effective framework for efficient 1,12-dodecanediol production.

**Table 2:** Comparison of biocatalytic production of C12 diols

Substrate (Initial conc., mM)	Product (Final conc., mM)	Genes	Yield (g/L)	Reaction time (h)	Productivity (g/L/h)^a, b^	Conversion rate (%)	Reactor	Reference
ω- Hydroxydodecanoic acid (10 mM)	1,12-Dodecanediol (9.7 mM)	MmCAR + Sfp + endogenous ALRs	1.97 g/L	15 h	0.131^a^	97%	Flask	(Ahsan et al. 2018)
ω- Hydroxydodecanoic acid (30 mM)	1,12-Dodecanediol (24.7 mM)	MmCAR + Sfp + GDH	5.0 g/L	24 h	0.208^a^	82%	Flask	(Ahsan et al. 2018)
Dodecanoic acid → ω-OHDDA (7mM )	1,12-Dodecanediol (6.7 mM)	CYP153A33 + CamAB → MmCAR + Sfp + GDH	1.40 g/L	15 h	0.093^a^	96%	Flask	(Ahsan et al. 2018)
Dodecane + 1-dodecanol (fed-batch, 120 g/L broth)	1,12-Dodecanediol (≈ 18.6 mM)	Reconstituted *CYP153A*(M.aq) operon + AlkL	3.76 g/L	71.5 h	0.055^b^	N.R. (Not Reported)	5 L bioreactor	(Hsieh et al. 2018)
1-Dodecanol (10 mM)	1,12-Dodecanediol (7.12 mM)	CYP153A33 P136A + CamA/CamB + FadL	1.44 g/L	5 h	0.288^a^	71.2%	Flask	(Park et al. 2022)
α,ω-Dodecanedioic acid (10 mM)	1,12-Dodecanediol (6.82 mM)	CAR–Sfp cell + ADH2–GroES/EL–DnaK/J/E cell	1.38 g/L	24 h	0.058^a^	68.2%	Flask	(Cha et al. 2021)
ω- Hydroxydodecanoic acid (10 mM)	1,12-Dodecanediol (9.77 mM)	MAB4714 CAR + Sfp + endogenous ADH	1.98 g/L	17 h	0.116^a^	97.7%	Flask	(Cha et al. 2021)
Dodecanoic acid methyl ester (100 mM)	1,12-Dodecanediol (27.0 mM)	PpAlkB + AlkG/T + FadL + MmCAR + Sfp	5.46 g/L	48 h	0.114^a^	27.0%	Flask	(Yoo et al. 2022)
Bio-based 1,12-diacid (fed-batch, 150 g/L broth)	1,12-Dodecanediol (≈ 336 mM)	MmCAR + Sfp + endogenous ADH/AKR	68.0 g/L	48 h	1.42^b^	≈ 100%	5 L fermenter	(Park et al. 2025)
1,12-Dodecanedioic acid (40mM)	1,12-Dodecanediol (27.7mM)	MAB4714 CAR + Sfp+yqhD+PPK2	5.60 g/L	1 h	5.6^c^	69.4%	Flask	This study

CAR, carboxylic acid reductase; Sfp, phosphopantetheinyl transferase; ALRs, aldehyde reductases; GDH, glucose dehydrogenase; CYP153A33, cytochrome P450 monooxygenase; CamAB, camphor 5-monooxygenase system (CamA: reductase, CamB: ferredoxin); AlkL, outer membrane protein; FadL, long-chain fatty acid transporter; ADH, alcohol dehydrogenase; AKR, aldo-keto reductase; GroES/EL and DnaK/J/E, molecular chaperones; PpAlkB, alkane hydroxylase; AlkG/T, rubredoxin/rubredoxin reductase system; PPK2, polyphosphate kinase 2

^a^ Whole-cell system: Productivity calculated based on the bioconversion time (reaction period only, excluding cultivation)

^b^ Fermentation system: Overall process productivity calculated based on the entire duration, including cell cultivation and product accumulation

^c^ Cell-lysate system: Productivity calculated based on the total bioconversion time

However, several challenges remain for scale-up of the cell-lysate platform. The primary limitation is the need for exogenous supplementation of costly cofactors such as NAD(P)H, ATP, and SHMP, and future work should incorporate dedicated cofactor recycling modules (e.g., GDH or FDH-based NAD(P)H regeneration) to improve controllability and reduce cofactor-related costs. Additionally, while wild-type enzymes provided a solid foundation for system-level optimization, future engineering of MabCAR and YqhD to enhance substrate specificity and catalytic efficiency may further improve overall productivity. Catalyst stability and reusability will also be important considerations for extended operation, motivating strategies such as immobilization or continuous-flow implementation. By addressing these scaling challenges, the high-rate platform developed in this study could provide a practical and economically feasible route for the rapid production of bio-based long-chain diols.

## Conclusion

In this study, the effects of ATP regeneration strategies, reaction system selection, and cofactor supply conditions on conversion efficiency were systematically investigated for the CAR-based biocatalytic production of 1,12-dodecanediol from 1,12-dodecanedioic acid. Whole-cell bioconversion experiments demonstrated that ATP availability is an important factor for C12 diol production, and the introduction of a PPK2-based ATP regeneration system enabled more efficient and sustained ATP supply compared with simple external ATP supplementation. Comparison of whole-cell and cell-lysate-based biocatalysis showed that both systems achieved similar final product titers. However, the cell-lysate system reached these titers much faster, resulting in higher time-based productivity. This acceleration may be attributed to the elimination of mass transfer limitations and the bypassing of cell membrane barriers, enabling high-rate bioconversion within 1–2 h compared to the 24–48 h typically required in whole-cell systems. Based on this result, subsequent cofactor effect analyses were conducted using the cell-lysate system. Under ATP regeneration conditions, Mg^2+^ concentration and redox cofactor supply were identified as key factors influencing conversion efficiency. When sufficient Mg^2+^ supply and ATP regeneration were ensured, NADPH and NADH resulted in comparable conversion efficiencies. This finding indicates that NADH, which is more favorable in terms of cost and availability, can serve as an effective alternative to NADPH in CAR-based C12 diol biocatalysis. This compatibility with NADH is particularly significant from an industrial perspective, as it allows for a substantial reduction in operating expenses and enhances the economic competitiveness of large-scale bio-based C12 diol production. These results provide a practical strategy for reducing cofactor related costs and improving the economic feasibility of C12 diol production processes. Specifically, a productivity of 5.60 g/L/h was achieved under optimized conditions, representing one of the highest reported values for flask-scale 1,12-dodecanediol production. Overall, this study highlights the importance of balancing ATP regeneration, reaction system selection, and cofactor supply conditions for achieving efficient C12 diol production. While further optimization regarding cofactor recycling and enzyme stability is required for successful industrial scale-up, our current expression system, based on the pCDFDuet-1::*ppk2* vector, was pre-designed with an additional multiple cloning site (MCS) to accommodate a NAD(P)H regeneration module. Future efforts will concentrate on the co-expression of a redox-regenerating enzyme, such as glucose dehydrogenase (GDH), within this vacant MCS to establish a more controllable and economically feasible dual-regeneration platform. These findings provide a promising foundation for the practical and economically feasible production of bio-based diols.

## Electronic Supplementary Material

Below is the link to the electronic supplementary material.

Supplementary Material 1

## Data Availability

All data generated or analyzed during this study are included in this published article.

## References

[CR1] Ahsan MM, Sung S, Jeon H, Patil MD, Chung T, Yun H (2018) Biosynthesis of medium-to long-chain α, ω-diols from free fatty acids using CYP153A monooxygenase, carboxylic acid reductase, and E. coli endogenous aldehyde reductases. Catalysts 8(1):4

[CR29] Akhtar MK, Turner NJ, Jones PR (2013) Carboxylic acid reductase is a versatile enzyme for the conversion of fatty acids into fuels and chemical commodities. Proc Nat Acad Sci, 110(1):87–92 23248280 10.1073/pnas.1216516110 PMC3538209

[CR31] Andexer JN, Richter M (2015) Emerging enzymes for ATP regeneration in biocatalytic processes. ChemBioChem 16(3):380–386 25619338 10.1002/cbic.201402550

[CR17] Basri RS, Rahman RNZRA, Kamarudin NHA, Ali MSM (2023) Carboxylic acid reductases: Structure, catalytic requirements, and applications in biotechnology. Int J Biol Macromol 240:124526 37080403 10.1016/j.ijbiomac.2023.124526

[CR23] Butler N, Kunjapur AM (2020) Carboxylic acid reductases in metabolic engineering. J Biotechnol 307:1–14 31628973 10.1016/j.jbiotec.2019.10.002

[CR18] Cao H, Li C, Zhao J, Wang F, Tan T, Liu L (2018) Enzymatic production of glutathione coupling with an ATP regeneration system based on polyphosphate kinase. Appl Biochem Biotechnol 185(2):385–395 29164506 10.1007/s12010-017-2664-4

[CR10] Cha TY, Yong Y, Park H, Yun HJ, Jeon W, Ahn JO, Choi KY (2021) Biosynthesis of C12 fatty alcohols by whole cell biotransformation of C12 derivatives using Escherichia coli two-cell systems expressing CAR and ADH. Biotechnol Bioprocess Eng 26(3):392–401

[CR16] Chen H, Zhang YHPJ (2021) Enzymatic regeneration and conservation of ATP: challenges and opportunities. Crit Rev Biotechnol 41(1):16–33 33012193 10.1080/07388551.2020.1826403

[CR44] Cheng F, Wang CJ, Gong XX, Sun KX, Liang XH, Xue YP, Zheng YG (2025) Assembly and engineering of BioBricks to develop an efficient NADH regeneration system. Appl Environ Microbiol 91(1):e01041–e01024 39660873 10.1128/aem.01041-24 PMC11784351

[CR39] CLARKE DM, Loo TW, GILLAM S, BRAGG PD (1986) Nucleotide sequence of the pntA and pntB genes encoding the pyridine nucleotide transhydrogenase of Escherichia coli. Eur J Biochem 158(3):647–653 3525165 10.1111/j.1432-1033.1986.tb09802.x

[CR34] Ding XW, Rong J, Pan ZP, Zhu XX, Zhu ZY, Chen Q, Zheng GW (2024) De novo multienzyme synthetic pathways for lactic acid production. ACS Catal 14(7):4665–4674

[CR4] Ensari ÖF, Alkan C (2025) 1, 12-dodecanediol among similar fatty alcohols as a phase change material for thermal energy storage. Solar Energy Adv 5:100079

[CR43] Garfinkel L, Garfinkel D (1985) Magnesium regulation of the glycolytic pathway and the enzymes involved. Magnesium 4(2–3):60–72 2931560

[CR25] Heuser F, Schroer K, Lütz S, Bringer-Meyer S, Sahm H (2007) Enhancement of the NAD (P)(H) pool in Escherichia coli for biotransformation. Eng Life Sci 7(4):343–353

[CR36] Hodgman CE, Jewett MC (2012) Cell-free synthetic biology: thinking outside the cell. Metab Eng 14(3):261–269 21946161 10.1016/j.ymben.2011.09.002 PMC3322310

[CR13] Horvat M, Winkler M (2020) In vivo reduction of medium-to long‐chain fatty acids by carboxylic acid reductase (CAR) enzymes: limitations and solutions. ChemCatChem 12(20):5076–5090

[CR2] Hsieh SC, Wang JH, Lai YC, Su CY, Lee KT (2018) Production of 1-dodecanol, 1-tetradecanol, and 1, 12-dodecanediol through whole-cell biotransformation in Escherichia coli. Appl Environ Microbiol 84(4):e01806–e01817 29180361 10.1128/AEM.01806-17 PMC5795085

[CR32] Kameda A, Shiba T, Kawazoe Y, Satoh Y, Ihara Y, Munekata M, Noguchi T (2001) A novel ATP regeneration system using polyphosphate-AMP phosphotransferase and polyphosphate kinase. J Biosci Bioeng 91(6):557–563 16233039 10.1263/jbb.91.557

[CR14] Karava M, Liang Q, van der Pol E, Winkler M, Kourist R (2025) Hydrogen-driven, ATP-dependent biocatalytic reduction of carboxylic acids under non-explosive conditions. Green Chem 27(47):15049–15055 41234477 10.1039/d5gc03751d PMC12605835

[CR9] Khusnutdinova AN, Flick R, Popovic A, Brown G, Tchigvintsev A, Nocek B, Yakunin AF (2017) Exploring bacterial carboxylate reductases for the reduction of bifunctional carboxylic acids. Biotechnol J 12(11):1600751 10.1002/biot.201600751 PMC5681412 28762640

[CR12] Kim YC, Choi H, Kim WY, Kim EJ, B. G., Yun H (2025) Metabolic engineering of Yarrowia lipolytica for enhanced microbial production of medium-chain α, ω-diols from alkanes via CRISPR-Cas9 mediated pathway optimization and P450 alkane monooxygenase overexpression. Front Bioeng Biotechnol 13:1695661 41209299 10.3389/fbioe.2025.1695661 PMC12588895

[CR40] Klompmaker SH, Kohl K, Fasel N, Mayer A (2017) Magnesium uptake by connecting fluid-phase endocytosis to an intracellular inorganic cation filter. Nat Commun 8(1):1879 29192218 10.1038/s41467-017-01930-5 PMC5709425

[CR30] Kramer L, Le X, Rodriguez M, Wilson MA, Guo J, Niu W (2020) Engineering carboxylic acid reductase (CAR) through a whole-cell growth-coupled NADPH recycling strategy. ACS Synth Biol 9(7):1632–1637 32589835 10.1021/acssynbio.0c00290

[CR22] Lee BW, Kim HT, Koh HG, Yu K, Kim G, Jung YJ, Park K (2025) Recombinant Escherichia coli-driven whole-cell bioconversion for selective 5-Aminopentanol production as a novel bioplastic monomer. Bioresources Bioprocess 12(1):58 10.1186/s40643-025-00904-6 PMC12149034 40490592

[CR28] Ling JG, Breuer HG, Stolterfoht-Stock H, Bakar FDA, Winkler M (2025) NADH driven enzymatic carboxylic acid reduction. ChemCatChem 17(9):e202401994

[CR6] Lu C, Weusthuis RA (2025) Microbial production of medium-chain-length diols: Current stage and perspectives. Bioresour Technol 435:132932 40609762 10.1016/j.biortech.2025.132932

[CR7] Lu C, Wijffels RH, Martins dos Santos VA, Weusthuis RA (2024) Pseudomonas putida as a platform for medium-chain length α, ω‐diol production: opportunities and challenges. Microb Biotechnol 17(3):e14423 38528784 10.1111/1751-7915.14423 PMC10963910

[CR35] Maier SK, Scherer S, Loessner MJ (1999) Long-chain polyphosphate causes cell lysis and inhibits Bacillus cereus septum formation, which is dependent on divalent cations. Appl Environ Microbiol 65(9):3942–3949 10473399 10.1128/aem.65.9.3942-3949.1999 PMC99724

[CR19] Motomura K, Hirota R, Okada M, Ikeda T, Ishida T, Kuroda A (2014) A new subfamily of polyphosphate kinase 2 (class III PPK2) catalyzes both nucleoside monophosphate phosphorylation and nucleoside diphosphate phosphorylation. Appl Environ Microbiol 80(8):2602–2608 24532069 10.1128/AEM.03971-13 PMC3993171

[CR41] Müller WE, Schröder HC, Wang X (2019) Inorganic polyphosphates as storage for and generator of metabolic energy in the extracellular matrix. Chem Rev 119(24):12337–12374 31738523 10.1021/acs.chemrev.9b00460 PMC6935868

[CR20] Neville N, Roberge N, Jia Z (2022) Polyphosphate kinase 2 (PPK2) enzymes: structure, function, and roles in bacterial physiology and virulence. Int J Mol Sci 23(2):670 35054854 10.3390/ijms23020670 PMC8776046

[CR37] Nocek BP, Khusnutdinova AN, Ruszkowski M, Flick R, Burda M, Batyrova K, Yakunin AF (2018) Structural insights into substrate selectivity and activity of bacterial polyphosphate kinases. ACS Catal 8(11):10746–10760

[CR8] Park H, Bak D, Jeon W, Jang M, Ahn JO, Choi KY (2022) Engineering of CYP153A33 with enhanced ratio of hydroxylation to overoxidation activity in whole-cell biotransformation of medium-chain 1-alkanols. Front Bioeng Biotechnol 9:817455 35059390 10.3389/fbioe.2021.817455 PMC8764613

[CR11] Park J, Jeon WY, Jang MJ, Lee HJ, Seo SH, Kim YS, Ahn JO (2025) An end-to-end microbial platform for 100% bio-based long-chain polyester: From renewable substrate to eco-friendly polymer. J Bioresour Bioprod

[CR21] Richardson KN, Black WB, Li H (2020) Aldehyde production in crude lysate-and whole cell-based biotransformation using a noncanonical redox cofactor system. ACS Catal 10(15):8898–8903 34306803 10.1021/acscatal.0c03070 PMC8294662

[CR24] Richter N, Neumann M, Liese A, Wohlgemuth R, Weckbecker A, Eggert T, Hummel W (2010) Characterization of a whole-cell catalyst co‐expressing glycerol dehydrogenase and glucose dehydrogenase and its application in the synthesis of l‐glyceraldehyde. Biotechnol Bioeng 106(4):541–552 20198657 10.1002/bit.22714

[CR38] Sauer U, Canonaco F, Heri S, Perrenoud A, Fischer E (2004) The soluble and membrane-bound transhydrogenases UdhA and PntAB have divergent functions in NADPH metabolism of Escherichia coli. J Biol Chem 279(8):6613–6619 14660605 10.1074/jbc.M311657200

[CR5] Schaffer S, Haas T (2014) Biocatalytic and fermentative production of α, ω-bifunctional polymer precursors. Org Process Res Dev 18(6):752–766

[CR3] Stempfle F, Ortmann P, Mecking S (2016) Long-chain aliphatic polymers to bridge the gap between semicrystalline polyolefins and traditional polycondensates. Chem Rev 116(7):4597–4641 27023340 10.1021/acs.chemrev.5b00705

[CR15] Strohmeier GA, Schwarz A, Andexer JN, Winkler M (2020) Co-factor demand and regeneration in the enzymatic one-step reduction of carboxylates to aldehydes in cell-free systems. J Biotechnol 307:202–207 31672531 10.1016/j.jbiotec.2019.10.016

[CR33] Sun C, Li Z, Ning X, Xu W, Li Z (2021) In vitro biosynthesis of ATP from adenosine and polyphosphate. Bioresour Bioprocess 8(1):117 10.1186/s40643-021-00469-0 PMC10992290 38650279

[CR42] Tang S, Liao D, Li X, Lin Y, Han S, Zheng S (2021) Cell-free biosynthesis system: Methodology and perspective of in vitro efficient platform for pyruvate biosynthesis and transformation. ACS Synth Biol 10(10):2417–2433 34529398 10.1021/acssynbio.1c00252

[CR26] Tavanti M, Hosford J, Lloyd RC, Brown MJ (2021) ATP regeneration by a single polyphosphate kinase powers multigram-scale aldehyde synthesis in vitro. Green Chem 23(2):828–837

[CR45] Yoo HW, Jung H, Sarak S, Kim YC, Park BG, Kim BG, Yun H (2022) Multi-enzymatic cascade reactions with Escherichia coli-based modules for synthesizing various bioplastic monomers from fatty acid methyl esters. Green Chem 24(5):2222–2231

[CR27] Yu K, Koh HG, Lee BW, Cha HG, Kim G, Jung YJ, Park K (2026) Comparative evaluation of l-theanine synthetases coupled with PPK2 based ATP regeneration under buffer-free and Mn2 + optimized conditions. Enzym Microb Technol 110834 10.1016/j.enzmictec.2026.110834 41762940

