# Supplementary material for: ATP regeneration-driven biocatalytic production of 1,12-dodecanediol with improved cofactor supply
Source: Bioresour Bioprocess. 2026 May 25;13(1):74. doi: 10.1186/s40643-026-01069-6 (PMC13199544; doi:10.1186/s40643-026-01069-6)
Supplement: Supplementary file 1 — Supplementary Material 1 [file 40643_2026_1069_MOESM1_ESM.docx]

**Supplementary File1.**

**ATP Regeneration-Driven Biocatalytic Production of 1,12-Dodecanediol with Improved Cofactor Supply**

**Yoon Jung Jung ^1^, Gaeul Kim ^1^, Kyungjae Yu ^1^, Byung Wook Lee ^1^, Jung Bin Shin ^1^, Jung-Oh Ahn ^2^, Si Jae Park ^3^, See-Hyoung Park ^1*^, Hyun Gi Koh ^1*^ and Kyungmoon Park ^1*^**

^1^Department of Biological and Chemical Engineering, Hongik University, Sejong, 30016, Republic of Korea

^2^Biotechnology Process Engineering Center, Korea Research Institute of Bioscience and Biotechnology, Ochang 28116, Republic of Korea

^3^ Department of Chemical Engineering and Materials Science, Graduate Program in System Health Science and Engineering, Ewha Womans University, Seoul, 03760, Republic of Korea

**^*^Correspondence:**

See-Hyoung Park: [shpark74@hongik.ac.kr](mailto:shpark74@hongik.ac.kr)

Hyun Gi Koh: [hgkoh@hongik.ac.kr](mailto:hgkoh@hongik.ac.kr)

Kyungmoon Park: [pkm2510@hongik.ac.kr](mailto:pkm2510@hongik.ac.kr)

**Supplementary Figure caption**

**Supplementary Figure S1.** Comparison of 1,12-dodecanediol production profiles in the absence and presence of glucose. Time-course production of 1,12-diol was evaluated using *E. coli* C cell lysate with 10 mM dodecanedioic acid as the substrate in the (a) absence and (b) presence of 1% (w/v) glucose. All reaction mixtures were commonly supplemented with 10 mM ATP, 2 mM NADPH, and 10 mM MgCl_2_.

**Supplementary Figure S2.** SDS-PAGE analysis of the soluble expression of target enzymes in *E. coli* C and *E. coli* CP. The soluble fractions of cell lysates were analyzed to verify the expression of target proteins.

**Supplementary Figure S3.** Preliminary screening of various carboxylic acid reductases (CARs) for 1,12-dodecanediol production. Maximum concentrations of 1,12-dodecanediol were compared using three different CAR candidates to evaluate their catalytic efficiency toward the dodecanedioic acid. The tested enzymes included Mab2962 and Mab4714 derived from *Mycobacterium abscessus*, and MsCAR derived from *Mycobacterium smegmatis*. The bioconversion was performed in a 5 mL reaction volume (pH 7.5, OD_600_ = 20) containing 10 mM dodecanedioic acid as the substrate. The reaction mixture was supplemented with 10 mM MgCl_2_, 10 mM ATP, 1% (w/v) glucose, and 2 mM NADPH.

**Supplementary Figure S1**

**
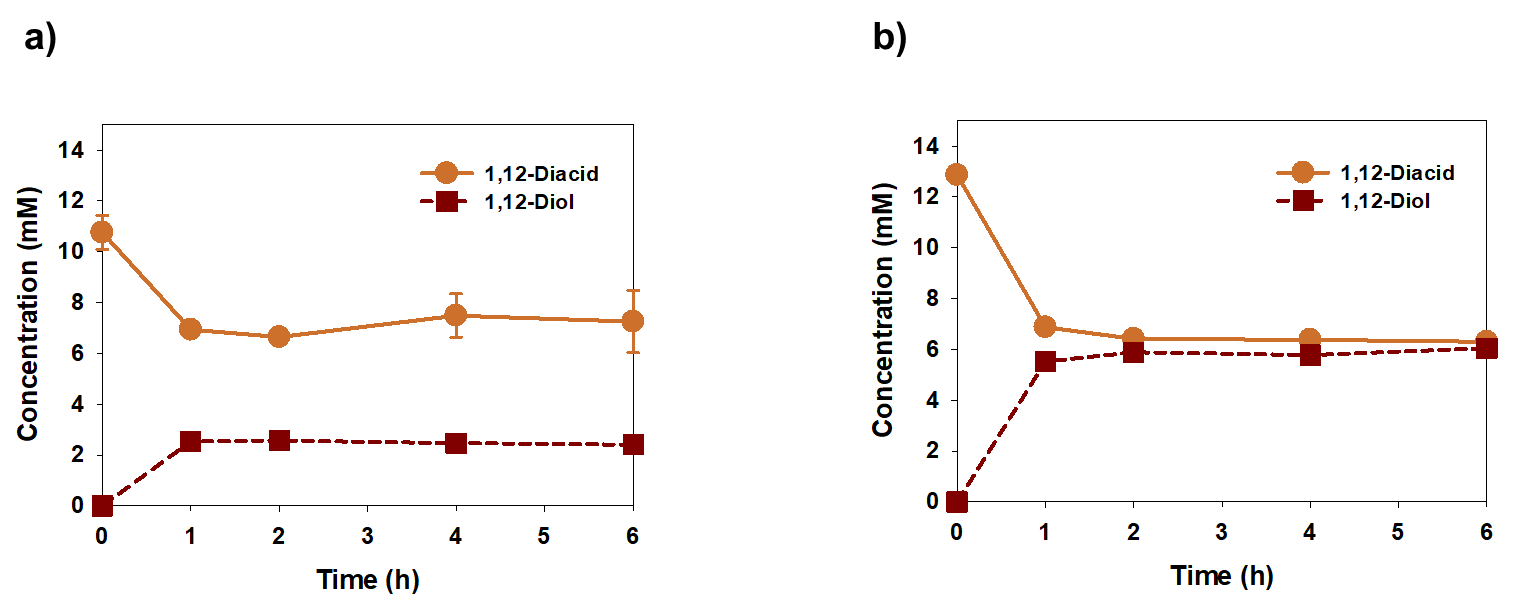
**

**Supplementary Figure S2**

**
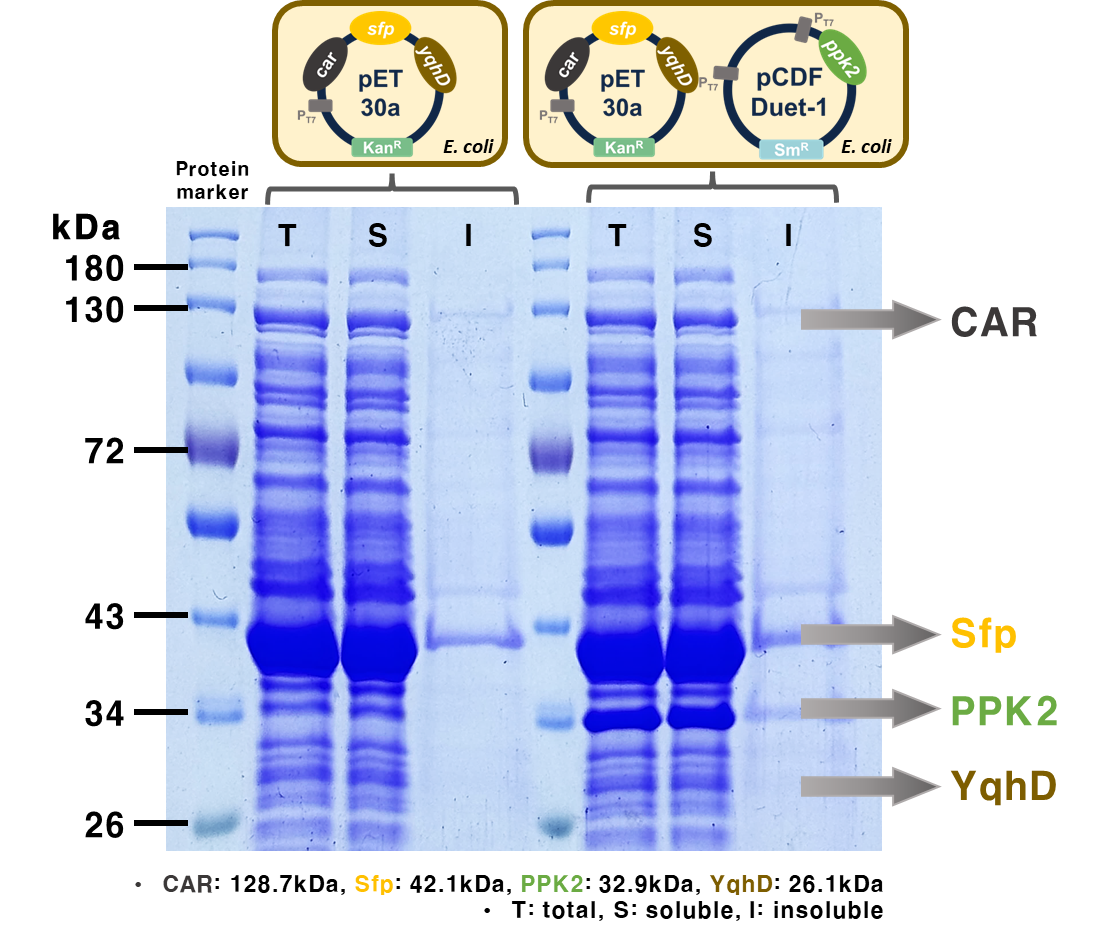
**

**Supplementary Figure S3**


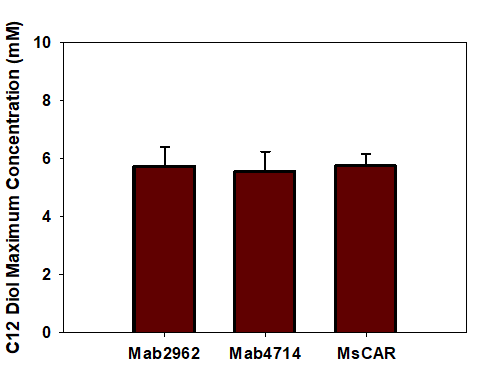


**Supplementary Table caption**

**Supplementary Table S1**. Nucleotide sequence of the *ppk2* gene and all primer sequences used in this study

**Supplementary Table S2**. Nucleotide sequence of CARs used in this study

**Supplementary Table S1**

| **Gene** | ***PPK2*** |
| --- | --- |
| **Nucleotide Sequence** | ATGATAAATATTTATAAGATAGATAAATTGAATAACTTCAACCTGAACAACCACAAAACCGATGATTACAGCCTGTGCAAAGACAAAGACACTGCGCTGGAACTTACCCAGAAGAATATCCAGAAGATCTATGACTACCAACAAAAGCTCTACGCTGAGAAGAAAGAAGGCCTGATCATCGCGTTCCAAGCGATGGATGCAGCAGGTAAAGACGGTACAATCAGAGAAGTGCTGAAGGCCTTGGCTCCGCAGGGTGTTCACGAAAAGCCGTTTAAAAGCCCGAGCTCAACGGAATTAGCGCATGATTACTTATGGCGTGTCCACAATGCTGTGCCGGAGAAGGGTGAAATCACCATTTTTAACCGTAGCCATTATGAAGACGTTCTGATTGGCAAAGTTAAAGAACTGTACAAATTCCAGAATAAGGCCGACCGTATTGATGAAAACACCGTGGTTGATAACCGCTACGAGGACATCCGTAATTTCGAGAAGTACTTGTACAATAACTCCGTGCGTATCATTAAAATCTTTTTGAACGTGTCGAAGAAGGAGCAGGCGGAGCGCTTCCTGAGCCGTATTGAGGAGCCGGAAAAGAACTGGAAATTTTCCGATAGCGACTTCGAAGAACGCGTTTATTGGGATAAATACCAGCAAGCGTTCGAGGACGCGATTAACGCAACCTCTACGAAGGACTGTCCGTGGTATGTTGTACCAGCGGACCGCAAATGGTATATGCGTTATGTCGTGTCTGAAATTGTCGTGAAGACCCTGGAGGAAATGAATCCGAAATACCCGACCGTTACCAAGGAGACGTTGGAGCGCTTTGAGGGCTATCGTACCAAACTGCTGGAAGAGTATAACTACGACCTGGATACCATCCGTCCGATTGAGAAGTGA |
| **Primers sequence (5’ to 3’)** | F: AAAAAA CATATG ATGATAAATATTTATAAGATAGA  R: AAAAAA CTCGAG TCACTTCTCAA |
| **Used enzyme** | F: NdeⅠ  R: XhoⅠ |
| **Reference** | This study |

**Supplementary Table S2**

| **Gene**  **(organism)** | **Nucleotide Sequence** |
| --- | --- |
| **Mab2962**  **(*Mycobacterium abscessus*)** | ATGACCGTGACCAACGAAACCAACCCACAGCAGGAGCAGCTATCCCGCCGTATTGAAAGTCTGCGCGAAAGCGATCCGCAGTTCCGGGCGGCCCAGCCCGACCCGGCGGTCGCCGAACAGGTGCTGCGCCCGGGCCTGCATCTTTCTGAAGCCATTGCGGCGTTGATGACTGGATACGCTGAGCGCCCGGCGCTCGGTGAGCGCGCACGCGAGTTGGTCACCGACCAGGATGGCCGCACCACGCTGCGCCTGTTGCCACGCTTCGACACCACCACATACGGCGAATTATGGTCCCGCACAACATCAGTCGCCGCTGCATGGCACCACGACGCCGCCCACCCGGTTAAGGCCGGCGATCTGGTGGCCACCCTGGGATTCACCAGCATCGACTACACCGTGCTGGATCTGGCGATCATGATCCTCGGTGGCGTGGCGGTTCCGCTACAGACCAGCGCCCCGGCTTCGCAGTGGACGACCATTCTGGCCGAAGCGGAACCCAACACTCTTGCGGTAAGCATCGAATTGATCGGCGCTGCAATGGAATCTGTGCGGGCCACGCCTTCCATCAAGCAGGTCGTCGTGTTCGACTACACCCCCGAGGTCGATGATCAACGGGAGGCATTCGAGGCAGCAAGCACACAACTCGCCGGCACCGGCATCGCCATTGAGACCCTCGATGCCGTCATCGCCCGCGGCGCCGCACTTCCGGCCGCACCGCTCTACGCACCATCGGCCGGCGACGATCCGCTGGCGCTGCTCATCTACACCTCCGGCAGCACCGGGGCTCCAAAGGGCGCCATGCACAGCGAAAACATCGTGCGCCGCTGGTGGATTCGTGAGGACGTCATGGCCGGCACCGAGAACCTGCCCATGATCGGGCTGAACTTCATGCCGATGAGTCACATCATGGGACGCGGCACCCTCACCTCCACCCTGTCTACCGGTGGAACCGGATACTTCGCGGCGTCCAGTGACATGTCAACGCTCTTCGAGGACATGGAGCTGATCCGCCCGACGGCCCTGGCCTTGGTTCCACGCGTGTGCGACATGGTGTTCCAGCGATTCCAGACCGAGGTGGACCGGCGTCTGGCGAGCAGCGACACCGCCAGTGCCGAGGCCGTTGCGGCCGAGGTCAAGGCCGATATCCGTGACAACCTCTTCGGTGGCCGCGTATCGGCGGTCATGGTCGGTTCTGCTCCGTTGTCCGAGGAGCTGGGTGAGTTCATCGAATCCTGCTTCGAGCTGAATCTGACCGATGGCTACGGCTCCACCGAAGCCGGCATGGTGTTCCGCGACGGCATCGTGCAACGCCCGCCGGTCATTGACTACAAGCTGGTTGACGTGCCCGAACTGGGCTACTTCTCCACCGACAAGCCGCACCCGCGCGGTGAGCTGCTGCTGAAGACCGACGGCATGTTCCTCGGGTACTACAAACGCCCCGAGGTGACTGCCGGCGTCTTCGACGCGGACGGTTTTTACATGACCGGCGACATCGTCGCCGAGCTGGCCCACGACAACATCGAGATCATCGATCGCCGCAACAACGTGCTCAAACTCTCACAGGGAGAGTTTGTCGCGGTCGCCACCTTGGAGGCCGAGTACGCCAATAGCCCTGTGGTGCACCAGATCTACGTCTACGGCAGCAGCGAACGGTCCTACCTGCTAGCAGTCGTGGTGCCGACGCCGGAGGCCGTGGCCGCCGCCAAGGGCGACGCGGCGGCACTCAAGACGACCATCGCGGACTCGCTGCAGGACATTGCCAAGGAGATCCAGCTGCAGTCCTACGAAGTCCCCCGTGACTTCATCATCGAACCGCAGCCATTCACCCAGGGCAACGGCCTGCTGACGGGTATCGCCAAGCTGGCGCGTCCGAACCTGAAGGCGCACTATGGACCGCGGCTGGAGCAGATGTACGCCGAAATCGCCGAGCAGCAGGCTGCCGAGCTTCGGGCGTTGCACGGAGTGGACCCAGACAAGCCCGCGCTGGAAACGGTCCTCAAGGCGGCGCAGGCCCTGCTCGGCGTCTCGTCGGCCGAACTGGCCGCGGACGCGCATTTCACCGATCTAGGTGGCGATTCGCTGTCCGCACTGTCCTTCTCGGATCTGCTGCGCGATATCTTCGCGGTCGAAGTACCGGTCGGAGTCATCGTCAGTGCCGCAAACGATCTCAGCGGTGTTGCGAAATTTGTTGATGAACAACGCTATTCGGGCGGGACGCGGCCGACCGCGGAGACGGTGCACGGCGCCGGGCATACGGAGATCCGGGCCGCGGACCTGACCCTGGATAAGTTCATCGACGAGGCCACCCTGCATGCGGCACCGTCGCTTCCGAAGGCCGTCGGGATCCCACACACCGTCCTGCTCACCGGGTCCAACGGCTACCTGGGCCACTACCTGGCACTGGAATGGCTTGAGCGCCTGGACAAGACAGAAGGCAAGCTGATCGCCATCGTCCGCGGTAAGAATGCCGAGGCCGCCTACCGCCGCCTCGAGGAAGCCTTCGACACCGGCGACACGCAGCTGTTGGCGCACTTCCGGTCGCTGGCCGACAAGCACCTCGAAGTACTGGCCGGCGATATCGGCGACCCCAACCTTGGCCTGGATGCCGACACCTGGCAGCGCCTGGCCGACACCGTCGACGTCATCGTGCACCCCGCCGCCCTGGTCAACCACGTACTGCCCTACAGCCAGCTGTTCGGACCGAATGTCGTCGGCACCGCCGAGATCATCAAGCTGGCCATCACTACCAAGATCAAGCCGGTCACCTACCTGTCCACGGTCGCGGTCGCGGCATATGTCGATCCGACGACATTCGACGAAGAGTCCGATATCCGGCTCATCAGCGCGGTGCGTCCCGTGGACGAGCTGTACGCGAACGGCTACGGCAACAGCAAGTGGGCCGGCGAGGTACTGCTGCGCGAAGCCCACGATCTGTGCGGACTACCCGTCGCGGTCTTCCGCTCCGACATGATCTTGGCCCACAGCCGCTACACCGGACAGCTCAACGTGCCCGACCAGTTCACCCGACTAATCCTCAGCCTCATCGCCACCGGAATCGCACCCGGCTCCTTCTACCAAGCACACGCCACCGGCGAACGCCCACTCGCCCACTACGACGGGCTACCCGGTGACTTCACCGCCGAGGCGATCACCACGTTGGGCACCCAGGTGGTCGACAGCTACGAGACCTACGACTGCGTGAACCCGCATGCAGACGGAGTCTCGCTGGACAACTTCGTCGACTGGCTCATCGAAGCCGGCTACCCCATCGCACGCATCGACAACTACACCGAATGGTTCACCCGCTTCGACACCGCCATCCGAAGCCTCCCCGAAAAACAGAAACAACACTCCCTACTACCACTGCTCCACGCATTCGAACAGCCGTCCGCCGCCGAGAACCACGGCGTCGTCCCGGCAAAGCGTTTCCAGCACGCTGTGCAGGCCGCCGGAATCGGTCCGGCCGGGCAAGACGGCACTACCGACATTCCCCACCTGTCGCGGCGGCTGATCGTGAAATACGCCAAGGACCTCGAACAGCTCGGACTCCTATGA |
| **Mab4714**  **(*Mycobacterium abscessus*)** | ATGACTGAAACGATCTCCACAGCGGCTGTCCCCACTACGGATCTCGAAGAGCAGGTGAAGCGACGCATCGAGCAGGTCGTGTCCAACGATCCGCAGCTGGCGGCGCTTCTCCCGGAAGATTCGGTCACCGAGGCGGTCAACGAGCCCGATCTACCGCTGGTCGAGGTGATCAGGCGACTGCTGGAGGGCTACGGTGACCGCCCGGCACTCGGCCAGCGCGCCTTCGAGTTCGTCACCGGGGACGACGGTGCGACCGTGATCGCGCTGAAGCCCGAATACACCACCGTCTCCTACCGCGAGTTGTGGGAACGTGCCGAGGCTATCGCTGCCGCGTGGCACGAGCAGGGCATCCGTGACGGCGACTTCGTCGCTCAGTTGGGTTTCACCAGCACGGACTTCGCGTCGCTCGACGTCGCGGGATTGCGTCTGGGCACCGTCTCGGTGCCCCTGCAGACGGGCGCGTCGCTGCAGCAGCGCAACGCGATTCTCGAAGAGACCCGGCCCGCAGTCTTTGCCGCGAGTATCGAATACCTTGATGCCGCCGTCGATTCGGTGCTTGCGACCCCCTCGGTGCGACTCCTCTCGGTTTTCGACTATCACGCGGAGGTCGACAGCCAGCGCGAAGCGCTGGAGGCTGTGCGGGCCCGGCTTGAGAGTGCCGGCCGGACGATCGTCGTCGAGGCCCTGGCGGAGGCTCTCGCGCGGGGGCGGGACCTGCCCGCCGCGCCGCTGCCCAGTGCAGATCCCGATGCCTTGCGTCTGCTCATCTACACCTCCGGCAGCACCGGTACCCCCAAGGGCGCCATGTATCCGCAATGGCTGGTCGCCAACTTGTGGCAGAAGAAGTGGCTCACCGACGATGTGATTCCGTCCATAGGCGTGAACTTCATGCCCATGAGCCACCTGGCGGGTCGCCTCACTCTCATGGGCACCCTTTCCGGTGGCGGAACCGCCTACTACATCGCTTCGAGCGATCTTTCGACTTTCTTCGAGGACATCGCGCTCATCCGCCCCTCCGAAGTGCTCTTCGTGCCGCGTGTGGTGGAGATGGTGTTCCAGCGTTTTCAGGCAGAATTGGACCGGTCCCTTGCCCCGGGTGAGAGCAACTCCGAGATCGCGGAGCGAATCAAGGTCCGCATCCGGGAACAGGACTTCGGCGGGCGTGTGCTCAGTGCTGGCTCCGGGTCGGCCCCGTTGTCTCCTGAGATGACGGAGTTCATGGAGTCGCTGCTGCAGGTGCCGTTGCGCGACGGGTATGGGTCCACCGAGGCCGGTGGTGTGTGGCGTGACGGAGTCCTGCAGCGTCCGCCCGTCACCGACTACAAGCTGGTTGACGTTCCGGAACTCGGATACTTCACCACAGATTCGCCGCATCCCCGTGGCGAGCTGCGGTTGAAGTCGGAGACGATGTTCCCCGGCTACTACAAGCGCCCGGAGACCACTGCCGATGTCTTCGATGACGAGGGGTACTACAAGACCGGTGACGTGGTCGCCGAGCTCGGGCCGGATCACCTCAAGTACCTCGACCGCGTCAAGAACGTCCTCAAGCTCGCGCAGGGAGAGTTTGTCGCGGTGTCAAAGCTGGAGGCCGCTTACACCGGCAGCCCGCTGGTCCGGCAGATCTTTGTGTACGGGAACAGTGAACGCTCGTTCCTGCTGGCTGTCGTGGTCCCGACACCCGAAGTCCTTGAGCGGTACGCAGATTCGCCAGATGCGCTCAAGCCCTTGATCCAGGATTCGCTGCAGCAGGTCGCCAAGGACGCGGAGCTGCAATCCTATGAGATACCGCGCGACTTCATCGTTGAGACGGTGCCGTTCACCGTCGAGTCCGGATTGCTATCGGACGCGCGAAAGCTGCTGCGCCCCAAGCTGAAGGATCACTACGGAGAGAGGCTGGAGGCGCTGTACGCCGAACTGGCGGAAAGCCAGAATGAGCGGCTGCGCCAGTTGGCCAGGGAGGCAGCCACGCGCCCGGTCCTGGAGACGGTGACCGATGCGGCCGCCGCGCTGCTGGGCGCATCGTCCTCGGATCTGGCTCCTGATGTGCGATTCATCGACCTCGGTGGCGACTCACTGTCGGCGCTGTCGTACTCCGAGCTGCTGCGCGACATCTTTGAGGTGGACGTTCCGGTGGGCGTCATCAACAGCGTCGCCAACGACCTTGCCGCGATCGCCCGGCACATCGAGGCGCAGCGGACCGGCGCCGCTACGCAGCCGACCTTTGCGTCGGTCCACGGCAAGGACGCGACGGTCATCACCGCCGGTGAACTCACCCTCGACAAGTTCTTGGACGAGTCACTGTTGAAAGCGGCCAAGGACGTTCAGCCGGCAACGGCCGATGTCAAGACCGTTCTAGTGACCGGCGGCAACGGCTGGTTGGGTCGTTGGCTGGTGCTCGATTGGCTGGAGCGGTTGGCACCCAATGGTGGCAAGGTCTACGCCCTCATTCGTGGCGCCGATGCCGAAGCAGCCCGGGCACGGTTGGACGCCGTGTACGAATCGGGTGATCCCAAGCTGTCCGCGCATTATCGTCAGCTGGCGCAACAGAGTCTGGAAGTTATCGCCGGCGATTTCGGCGACCAGGATCTCGGTCTATCCCAGGAAGTTTGGCAGAAGCTGGCCAAGGACGTGGACCTGATCGTGCACTCCGGTGCCTTGGTGAACCACGTGCTGCCGTACAGCCAGTTGTTCGGTCCGAATGTGGCGGGTACCGCCGAGATCATCAAGCTGGCAATTTCGGAGCGGCTCAAGCCGGTCACCTACCTGTCGACGGTGGGCATCGCCGACCAGATTCCGGTGACGGAGTTCGAGGAAGACTCCGATGTTCGTGTGATGTCGGCCGAGCGCCAGATCAATGACGGCTACGCGAACGGATACGGCAACTCAAAATGGGCCGGCGAGGTGCTGTTGCGGGAGGCTCATGACCTAGCGGGGCTGCCGGTGCGTGTGTTCCGCTCCGACATGATCCTGGCGCACAGTGACTACCACGGACAGCTCAACGTCACCGACGTGTTCACCCGGAGCATCCAGAGTCTGCTGCTCACCGGTGTTGCACCGGCCAGCTTCTATGAATTGGATGCCGACGGCAATCGGCAGCGCGCTCACTATGACGGTGTGCCCGGCGATTTCACCGCCGCATCGATCACCGCCATCGGCGGTGTGAACGTGGTAGACGGTTACCGCAGCTTCGACGTGTTCAACCCGCACCATGACGGTGTCTCGATGGATACCTTCGTCGACTGGCTGATCGACGCAGGCTACAAGATCGCGCGGATCGACGATTACGACCAGTGGCTCGCCCGGTTCGAGCTGGCCCTCAAGGGATTGCCCGAGCAGCAGCGGCAACAGTCGGTGTTGCCACTTCTCAAGATGTACGAGAAGCCGCAACCGGCGATCGACGGAAGTGCACTTCCGACCGCAGAATTCAGTCGCGCCGTGCACGAGGCGAAGGTCGGAGACAGCGGTGAGATACCGCACGTCACCAAGGAGCTGATCCTCAAGTACGCCAGCGATATTCAGCTGTTGGGCCTGGTGTAG |
| **MsCAR**  **(*Mycobacterium smegmatis*)** | ATGACGGCTGGTGCGGCGGCTCGCGTTGCCAAACTGTTCGAGTCCGATCCCCAATTCCGGGCAGCCATGCCGGATCCAGCGGTGATGGACTCGCTGCTGGCGCCCGGCCTGCGTTTATCCCAGGTACTCCACGCGTTGCTCAGCGGTTACGCGGAGCGCCCGGTGATGGGTTTCCGGTCCCGCGAGTCGGTGGTCGACACCGCCACCGGCCGCACGGTCGACCGGCTGCTCCCTGCCTTTGAAACCATCACCTATGGGCAACTCCTGGAAGACATCTCGGCCATCCTCGCGGAGTGGCAGCATGGCGACATTCCCATGGGCGCCGGCGACTTCATCGCCACCATCGGCTTCTCCAGTCCCGACTACGTCACCCTGGATCTGGCCACCCTCATGAATGGTTCGGTCTCGATCCCACTGCAGCACAACACATCTGTGGCGCAGCTGCGGATGATGCTGGAGGAGACCAGCCCACGCCTGGTGGCGGCGAGCGCGGACTGCCTGGATCTCGCGGTCGAGGCAGCTGTCGGGCTTACCGATCTGCGACGGGTTGTGGTGTTCGATTACCGCGCCGAGACCGACGATCATCGCGAAAAACTGGCCACGGCAAGAGAACGCTTGCACGCGGCCGGTATGGACGTTGTAGTCGAACCGCTCGCAGAGGTGATCGGGAGAGGACGAGACCTACCCGAACCCGTGCTGTACACGGCCGGGGACGATCAGCGCACGGCCCTGATCATGTACACCTCCGGTAGCACCGGCGCGCCCAAGGGGGCGATGTTCACCGAGTGGACGGTGACCCGCTTCTGGTCCTCGGGCGCCGCCCCCAACCGGGACACCCCGATCATCAACGTGAACTTCCTGCCGCTCAACCACCTTGCGGGCCGGGTAGGACTGCTGACGGCCTTCATTCCCGGCGGCACATGCTACTTCGTCCCCGAGAGCGATCTGTCCACCCTGTTCGAGGACTGGCAGCTGGCACGGCCCACCCATATGGGTGTGGTTCCCCGTGTCGTCGACATGCTCTTCCAGCACTACCAAACGCGAGTGGACGCACTGATGGCCGGGGGAACCGACGTCGACACCGCCGATCGGCTAGCCAAAACCGAACTGCGCGAAGATGTCCTGGGCGGGCGTGTGGTCGCCGGCATGCTCGCCACCGCGCCGTTGTCCCCCGAGATGAAGGCTTTCCTGGAGTCCTCATTGGACTTTCATCTGCTTGATCTGTACGGCCTGACCGAGGTCGGCGGCGTGTTCCGAGACGGCAAGATTTCCCGGCCGCCGGTGCTCGACTACAAGCTCGTCGATGTTCCTGAGCTCGGGTACTACACCACCGACAAGCCCCATCCGCGTGGCGAATTGCTGGTCAAGAGTGCCACCGCAACGCCCGGCTACTACAAGCGTCCCGACGTCACCGCCGAGGTGTTCGACGCCGATGGCTACTACCGCACGGGCGATGTCATGGCGGAGGTCGCGCCGGACCAATTGGTGTACGTGGACAGGCGCAATAACGTCATCAAGCTCGCCCAGGGCGAGTTCGTCGCGGTCGCCAATTTGGAAACGGTCTATGTGGGTGCGCCGCTGGTGCGCCAGATCTTCGTCTACGGCAACAGCGAACGCGCATACCTCCTCGCCGTTGTGGTGCCCACCGAGGAAGCCCTGCGGGCACACCCGGACCCCGTCGAACTGAAGAATTCGATCCGGGAGTCACTGCAGCGGACCGCCCGCTCCAACCACCTGCATTCCTACGAGCTGCCCGCCGACTTCATTATCGAAACCACTCCATTCACGATCGAGAGTGGGATGCTTGCGGCTGTCGGTAAGCCGATACGTCCCAAGATGATCGAGCACTACGGCGACCGGCTCGAGCAGCTCTACGTCGACCTCGCCGAGGCACGCGTCCAGGAACTGCGGCAGCTCCGCGATACGGCGCAACAACGCCCGGTCCTCGATACCGTCACCGAGGCCGCCCAGGCCCTCCTCGGCATGTCTGCGGACGCCGTCCGTCCCGACCACCACTTCATCGACCTCGGCGGAGATTCGCTGTCCGCGTTGACATTCTCCAATCTTCTTCGAGACCTCTTCGACGTCGAGGTTCCGGTCGGTGTGATCACCGGCCCGGCGGCCGATCTGCGCAAGCTCGCCGCTTACATCCAGCACGAACGGGAGCACAGCACCGCGACCGCTGCCAGCGTGCACGGGCTCGACACCACCGTCATCAGCGCCACCGAACTGACACTCGACAAGTTCATCGACGCCGAGACACTCCACAACGCTTCGCAACTCGACGTGCCGGCGGGCGCGGTAGCTACCGTTCTGCTCACCGGCGCCAACGGATATCTCGGAAGATTCCTCTGCCTGGAGTGGCTGCAACGGCTGTCCCAGACAGGTGGACAACTGATCTGCCTGGTCCGCGGCGACAACGCCGATCAAGCCCTCGCGCGCCTCGTTGCCGCCTACGGCGACACCGATCGCACACTGCTCGAGGAGTTCCACACCCTGGCTCGACGGCACCTGCGCGTGATCGCCGCCGATATCGCTCAGCCGCGCTTCGGCGTGGATGACGCCACCTGGGAGCAGCTGGCCCGCGATGTCGACAAGATCGTGCATCCGGCCGCGCTGGTCAACCACGTGCTGCCCTACAACCAGCTGTTCGGCCCCAATGTGTTTGGCACGGCGGAGGTTATCCGGCTGGCCCTGACCACCCGGATCAAGCCGGTGACCTATCTGTCGACGATGGCCGTGGCCATGACCGTGCCCGATTTCGACGAGGACGGGGACATCCGCACGGTGAGTCCCACCCGGCATATCGACCCCGGCTACGCCAACGGGTACGCCAACAGCAAATGGGCCGGCGAGGTGCTGCTGCGGGAGGCACACGACATATGCGGCCTGCCGGTCAGCGTGTTCCGGTCCGACATGATCCTGACGCACCGCCGTTACAGCGGACAACTCAACGTCACCGACGCCTTCACCCGCATGCTGCTGAGCCTGGTGCTCACCGGCATCGCGCCGCGAAGCTTTTACCAAGGCGATGGCAGCGGTGCCCGCCCACGCGCTCACTACGAGGGGCTGCCGGTCGATTTCGTCACCGAAGCCATTACCAGCCTCGGCCTGTCCTCGTCCGAGGGATTTCGCTCGTACGACGTCATGAATCCTCACGATGACGGCATTTCTGTGGACACCTTTGTCGACTGGCTCATGGAAGATGGGCATTCCATCGACATCATCGACAACTACGACGAATGGCTGTCCCGTTTCGAGACGGCATTGCGAGGTCTGCCCGACGAGCAGCGGCGCGCCTCAGTACTTCCGCTCCTCGATGCGTATCGGATACCGGGCAACCCGCGCCGTGCTGCCGCCACGCCCAATCATGTATTCCGGAAAGCCGTACAGGAGAACAACATCGGAGGTGACGGCGCCGATATTCCGCAAATCGATCGTGCGCTGATCGCCAAATACATCGCCGATCTACGAGCACACAGGCTGCTGTGA |
